# JAK/STAT3 represents a therapeutic target for colorectal cancer patients with stromal-rich tumors

**DOI:** 10.1186/s13046-024-02958-4

**Published:** 2024-03-01

**Authors:** Kathryn A. F. Pennel, Phimmada Hatthakarnkul, Colin S. Wood, Guang-Yu Lian, Sara S. F. Al-Badran, Jean A. Quinn, Assya Legrini, Jitwadee Inthagard, Peter G. Alexander, Hester van Wyk, Ahmad Kurniawan, Umar Hashmi, Michael A. Gillespie, Megan Mills, Aula Ammar, Jennifer Hay, Ditte Andersen, Colin Nixon, Selma Rebus, David K. Chang, Caroline Kelly, Andrea Harkin, Janet Graham, David Church, Ian Tomlinson, Mark Saunders, Tim Iveson, Tamsin R. M. Lannagan, Rene Jackstadt, Noori Maka, Paul G. Horgan, Campbell S. D. Roxburgh, Owen J. Sansom, Donald C. McMillan, Colin W. Steele, Nigel B. Jamieson, James H. Park, Antonia K. Roseweir, Joanne Edwards

**Affiliations:** 1 School of Cancer Sciences, Wolfson Wohl Cancer Research Centre, University of Glasgow, Glasgow, G61 1QH UK; 2 Department of Surgery, Glasgow Royal Infirmary, Glasgow, G31 2ER UK; 3 University of Glasgow Medical School, Glasgow, G12 8QQ UK; 4 CRUK Scotland Institute, Glasgow, G61 1BD UK; 5 Glasgow Tissue Research Facility, Queen Elizabeth University Hospital, Glasgow, G51 4TF UK; 6 Bioclavis Ltd, Glasgow, Queen Elizabeth University Hospital, Glasgow, G51 4TF UK; 7 CRUK Clinical Trials Unit, The Beatson West of Scotland Cancer Centre, Gartnavel Hospital, Glasgow, G12 0XH UK; 8 Wellcome Centre for Human Genetics, University of Oxford, Oxford, OX3 7BN UK; 9 NIHR Oxford Biomedical Research Centre, Oxford University Hospitals NHS Foundation Trust, John Radcliffe Hospital, Oxford, OX3 9DU UK; 10 Edinburgh Cancer Research Centre, IGMM, University of Edinburgh, Crewe Road, Edinburgh, EH4 2XU UK; 11 The Christie NHS Foundation Trust, Manchester, M20 4BX UK; 12 Southampton University Hospital NHS Foundation Trust, Southampton, SO16 6YD UK; 13 Department of Pathology, Queen Elizabeth University Hospital, Glasgow, G51 4TF UK; 14 Department of Surgery, Queen Elizabeth University Hospital, Glasgow, G51 4TF UK

**Keywords:** Colorectal cancer, Cellular signaling, JAK/STAT3 signal transduction, Tumor microenvironment, Prognosis, Spatial biology, JAK inhibitors, Tumor-stroma, Patient-derived organoids, Stratified medicine, Biomarkers

## Abstract

Colorectal cancer (CRC) is a heterogenous malignancy underpinned by dysregulation of cellular signaling pathways. Previous literature has implicated aberrant JAK/STAT3 signal transduction in the development and progression of solid tumors. In this study we investigate the effectiveness of inhibiting JAK/STAT3 in diverse CRC models, establish in which contexts high pathway expression is prognostic and perform in depth analysis underlying phenotypes. In this study we investigated the use of JAK inhibitors for anti-cancer activity in CRC cell lines, mouse model organoids and patient-derived organoids. Immunohistochemical staining of the TransSCOT clinical trial cohort, and 2 independent large retrospective CRC patient cohorts was performed to assess the prognostic value of JAK/STAT3 expression. We performed mutational profiling, bulk RNASeq and NanoString GeoMx® spatial transcriptomics to unravel the underlying biology of aberrant signaling. Inhibition of signal transduction with JAK1/2 but not JAK2/3 inhibitors reduced cell viability in CRC cell lines, mouse, and patient derived organoids (PDOs). In PDOs, reduced Ki67 expression was observed post-treatment. A highly significant association between high JAK/STAT3 expression within tumor cells and reduced cancer-specific survival in patients with high stromal invasion (TSP^high^) was identified across 3 independent CRC patient cohorts, including the TrasnSCOT clinical trial cohort. Patients with high phosphorylated STAT3 (pSTAT3) within the TSP^high^ group had higher influx of CD66b + cells and higher tumoral expression of PDL1. Bulk RNAseq of full section tumors showed enrichment of NFκB signaling and hypoxia in these cases. Spatial deconvolution through GeoMx® demonstrated higher expression of checkpoint and hypoxia-associated genes in the tumor (pan-cytokeratin positive) regions, and reduced lymphocyte receptor signaling in the TME (pan-cytokeratin- and αSMA-) and αSMA (pan-cytokeratin- and αSMA +) areas. Non-classical fibroblast signatures were detected across αSMA + regions in cases with high pSTAT3. Therefore, in this study we have shown that inhibition of JAK/STAT3 represents a promising therapeutic strategy for patients with stromal-rich CRC tumors. High expression of JAK/STAT3 proteins within both tumor and stromal cells predicts poor outcomes in CRC, and aberrant signaling is associated with distinct spatially-dependant differential gene expression.

**Supplementary Information:**

The online version contains supplementary material available at 10.1186/s13046-024-02958-4.

## Introduction

Colorectal cancer (CRC) remains a leading cause of cancer-related mortality worldwide. Existing treatment relies on surgery with the use of perioperative chemotherapy/chemoradiotherapy as adjuncts. However, given the highly heterogenous nature of CRC, recent research has focused on employing a precision medicine approach to identify targeted therapies against molecularly segregated CRC. This has been successful in a small subgroup of patients with advanced stage mismatch repair (MMR) deficient disease through the adoption of immune checkpoint inhibitors [1].

The development of the consensus molecular subtypes (CMS) and cancer cell intrinsic subtypes (CRIS) for CRC in the last decade have enabled subtyping of patients with distinct differences in the tumor biology and prognosis [1–3]. The identification of more clinically translatable histology-based subtyping methods including Tumor Stroma Percentage (TSP) and Glasgow Microenvironment Score (GMS) have been useful tools in identifying independently prognostic phenotypes [2, 4–6]. The establishment of novel and repurposed therapeutics best applicable to each subtype remains a key area of research. There is a profound association between high TSP and poor outcome in CRC, which has been extensively validated. However, the mechanisms underlying this are poorly understood [7].

In malignancy overexpression of the transcription factor signal-transducer and activator of transcription 3 (STAT3) has been identified in ovarian, pancreatic, gastric, and lung cancer and associated with poor patient outcomes [8–11]. Hyperactivation of the pathway is linked to many hallmarks of cancer including epithelial to mesenchymal transition (EMT), proliferation, angiogenesis, and metastases [12, 13]. These data suggest that STAT3 could be a promising therapeutic target for CRC patients, and JAKi could be repurposed for use in combination with existing therapies in CRC. However, it is not yet clear if there is a specific subset of CRC patients likely to respond to pathway inhibition.

We hypothesise that histological changes observed in high TSP cases could be underpinned by dysregulation of targetable cellular signaling pathways such as JAK/STAT3. Signal transduction is canonically initiated by IL6 binding membrane bound or soluble IL6R, which causes activation of one of four JAK proteins (JAK1, JAK2, JAK3, TYK2) within the cell [14, 15]. This ultimately leads to phosphorylation of STAT3 at tyrosine 705, whereby STAT3 homodimerizes and translocates to the nucleus to act as a master regulator of cancer-promoting genes [14]. STAT3 can become maximally activated through phosphorylation at serine 727 [16]. Janus kinase inhibitors (JAKi), which prevent activation of STAT3 are already used in the clinic for patients with myeloproliferative disorders [17].

In this study we aim to elucidate the therapeutic potential of inhibiting signal transduction using 3 repurposed JAKi in recapitulative disease models. We subsequently aim to establish a prognostic relationship of JAK/STAT3 expression relative to TSP in 3 retrospective CRC cohorts complemented by multi-omic interrogation of the impact of JAK/STAT3 overexpression.

## Material and methods

### Cell lines

Cell lines utilized included DLD-1, HT29, SW620, HCT116, SW837 and CCD18Co which were obtained through American Type Tissue Culture (ATCC) and cultured according to guidelines. For detection of pSTAT3^tyr705^, a solid phase sandwich ELISA kit was utilized (DYC4607B-2, R&D Systems, Minneapolis, MN, USA). Samples were prepared in 96 well plates and conditioned medium harvested 48 h post-treatment. For WST-1 cell viability assays, cells were plated, left to adhere overnight, and treated using a range of concentrations of Ruxolitinib (#S1378), Tofacitinib (#S5001) or AZD1480 (#S2162) for 72 h (Selleckchem, Houston, TX, USA). WST-1 reagent (#11,644,807,001, Roche, Basel, Switzerland) was added for 1 h and absorbance read at 450 nm using a TECAN Infinite PRO (TECAN Group Ltd, Zurich, Switzerland). Treated sample data were normalized to vehicle control samples. All experiments were performed to *n* = 3. Cells were tested for mycoplasma before commencing experiments.

### Silencing of STAT3

To knockdown STAT3 in HCT116 cells, scramble sequence or si-RNA for STAT3 (4,390,844, Invitrogen, Waltham, MA, USA) was transfected into the cells with lipofectamine RNAiMAX (13,778,100, Invitrogen, Waltham, MA, USA) for 24 h before the cells were used for cell viability assay.

### Mouse organoids

Organoid lines derived from *VillinCre*^*ER*^*; Kras*^G12D/+^; *Trp53*^fl/fl^; *Notch1*^TG/+^ (KPN) and *VillinCre*^*ER*^*; Apc*^*fl/*+^*; Kras*^*G12D/*+^*; Trp53*^*fl/fl*^*;TgfbrI*^*fl/fl*^ (AKPT) mouse models (1 line each) were obtained from the Sansom Laboratory [18]. The experiment was performed according to UK Home Office regulations (Project Licenses 70/8646, PP3908577, 60/4183) and was reviewed by local animal welfare and an ethical review committee at the University of Glasgow. Mice were housed in conventional cages at constant temperature (19–23 °C) and humidity (55% ± 10%) under a 12-h light–dark cycle and were allowed access to standard diet and water ad libitum. A KPN male mouse on a C57BL/6J (generation N8) background of 8 weeks of age were induced with a single intraperitoneal injection of 2mg tamoxifen and aged until clinical endpoint as evidenced by anemia, hunching and/or weight loss to generate small intestinal tumor organoid lines as previously described [18]. Intracolonic induction in a 12-week-old female mouse *villin*Cre^ER^ *Apc*^fl/+^ *Kras*^G12D/+ ^*Trp53*^fl/fl ^*Tgfbr1*^fl/fl^ on a C57BL/6 background (generation N6/N7) was performed under general anesthesia. Three 70µl 100uM dose of 4-hydroxy tamoxifen (H7904-5MG from Sigma) were injected from the mid-colon to distal colon into the sub-mucosa via a colonoscope (Karl Storz TELE PACK VET X LED endoscopic video unit). At clinical endpoint (weight loss with the presentation of hunching) colonic tumor tissue was collected from a single tumor that had grown and organoid cell lines were generated as previously described [18]. KPN and AKPT tumors present as CMS4 disease. Organoids were cultured as described in supplementary data 2 [16].Organoids were grown in advanced DMEM was supplemented with B27 (12,587,001, Thermo Fisher Scientific, Waltham, MA, USA), N2 (17,502,001 Thermo Fisher Scientific, Waltham, MA, USA), murine Noggin (250–38, Peprotech, London, UK) and EGF (AF-100–15, Peprotech, London, UK). Organoids were grown in 20µL domes of Cultrex® type R1 basement membrane extract (BME) (R&D Systems, Minneapolis, MN, USA) in 6 well plates. For WST-1 assays organoids were grown in 5µL domes in 96 well plates and assays were completed as above. IF staining post-drug treatment for Caspase 8 (NB100-56116, Novus Biologicals, Abingdon, UK), and Ki67 (14–5698-80, Invitrogen, Waltham, MA, USA) was performed as previously described (20).

### Patient-derived organoids (PDOs) and explants

Establishment and culture of PDOs was performed as previously described [17]. For drug screening experiments tumor PDOs were seeded as fragments in 5μL Matrigel (Corning Inc, Corning, NY, UCA) in 96 well plates and grown for 48 h using IntestiCult™ human organoid medium (#06010, Stemcell Technologies, Vancouver, Canada) supplemented with RHO/ROCK pathway inhibitor (72,308. Stemcell Technologies, Vancouver, Canada). Medium was removed and replaced with either fresh medium, 0.1% DMSO, 1 µM-100 µM Tofacitinib, 1 µM-100 µM Ruxolitinib or 1 µM-100 µM 5-fluouracil (5FU) in triplicate. Plates were incubated for 72 h at 37 °C 5% CO_2_. WST-1 reagent (#11,644,807,001, Roche, Basel, Switzerland) was added, plates were incubated at 37 °C 5% CO_2_ for 2 h and absorbance read at 450 nm using a TECAN Infinite PRO (TECAN Group Ltd, Zurich, Switzerland). After drug treatments, 3 patient-derived organoid Sanger lines (25, 31, 37) were expanded sufficiently to perform IF staining for markers of mid-phase apoptosis (Caspase 8, NB100-56116, Novus Biologicals, Abingdon, UK) and proliferation (Ki67, #M7240, Agilent Technologies Santa Clara, CA, USA). IF staining was performed as previously outlined (20).

Explants were derived from CRC and adjacent normal tissue from surplus resection tissue from patients undergoing surgery with curative intent within Greater Glasgow and Clyde NHS hospitals through the Glasgow Biorepository (ETHICS). Tissue was chopped into small pieces (3 mm) and place in DMEM overnight at 5%CO_2_ 37 °C. Medium was replaced after 24 h with vehicle control, Ruxolitinib or Tofacitinib containing medium and left for 48 h. Explants were washed in PBS and fixed ion 4% PFA for 1 h before being transferring to ethanol and embedded into paraffin blocks. Sections were cut at 4 μM thickness and put onto glass slides. Sections were stained via IHC for Ki67 (1:150) (Agilent Technologies Dako), MHCI (1:400) (BNB120-6405 Novus Biologicals, Littleton, Ontario, CA) using a Leica Bond Rx (Leica Biosystems, Wetzlar, Germany).

### Colorectal cancer clinical cohorts

We investigated 3 independent CRC resected patient cohorts, including the TransSCOT clinical trial cohort, with patient characteristics outlined below and conformed to REMARK guidelines.

### Cohort 1: Glasgow combined array (ethical approval WS/16/0207)

Cohort 1 consisted of 1030 CRC patients who underwent surgery with curative intent in Glasgow Royal Infirmary, Western Infirmary or Stobhill hospitals, Glasgow between 1997 and 2007. Patients were graded using the 5th edition of TNM staging. The cohort comprised patients with stage I-IV disease. In cohort 1 32% (*n* = 230) patients were < 65 years of age and 68% (*n* = 494) were > 65 years of age with a median age of 69. This cohort consisted of 49% female and 52% male patients. Clinical follow up data were last updated in 2017 from NHS Greater Glasgow and Clyde Safe Haven data. At this time, 324 patients (32%) had died of primary colorectal cancer, 332 patients (33%) had died of other causes and 355 patients (35%) were still alive. Cancer-specific survival (CSS), (date of surgery until last follow up) was used as a clinical endpoint throughout this study. Mean follow up time was 139 months.

### Cohort 2: The TransSCOT clinical trial cohort [19] (ethical approval 07/S0703/136 until 2019, 16/WS/0207 2019 onwards)

In addition to retrospective cohorts, we analysed the prospective TransSCOT clinical trial cohort (cohort 2) [19]. The TransSCOT clinical trial cohort consisted of 2912 patients from the SCOT clinical trial. Patients were graded using the 7th edition of TNM staging. The cohort consisted of stage III and high-risk stage II patients (≥ 1 of T4 disease, tumor obstruction with or without preoperative perforation, < 10 lymph nodes harvested, poor tumor differentiation perineural invasion or extramural venous invasion or lymphatic invasion). In the TransSCOT cohort 54% of patients were < 65 years of age. Patients were randomly assigned to a treatment arm, with both CAPOX (capecitabine and oxaliplatin) and FOLFOX (bolus and infused fluorouracil with oxaliplatin) regimens utilized over 3- or 6-month duration. The clinical outcome measure used for cohort 3 was disease-free survival (DFS) (date of surgery/randomisation until date of recurrence or all-cause mortality). Patients were followed up for at least 3 years and at this time there were 2221 (76%) patients alive and 691 (24%) patients who had died of cancer. The mean follow-up time was 35 months.

### Cohort 3: Glasgow royal infirmary array (ethical approval MREC/01/0/36)

Cohort 3 consisted of 784 CRC patients who underwent surgery with curative intent within Greater Glasgow and Clyde health board between 1997–2013. This cohort comprised patients diagnosed with stage II-IV disease and clinical outcome was measured via CSS. In this cohort 33% (*n* = 233) patients were < 65 and 67% (*n* = 484) patients were > 65 years of age at the time of surgery. Of the patients included 45% were female and 55% were male. Follow up was updated in 2020 from NHS Greater Glasgow and Clyde Safe Haven. At this time 275 (35%) were alive, 231 (30%) had died of primary cancer and 277 (35%) had died of other causes. Mean follow up time was 89 months.

### Immunohistochemical assessment of JAK1, JAK2, STAT3, pSTAT3^tyr705^ and pSTAT3^ser727^

Immunohistochemical staining was performed to detect IL6R (#ab128008, Abcam, Cambridge, UK), JAK1 (#3344, Cell signaling, Danvers, MA, USA), JAK2 (#3773, Cell signaling, Danvers, MA, USA), STAT3 (#9132, Cell signaling, Danvers, MA, USA), pSTAT3^tyr705^ (#9131, Cell signaling, Danvers, MA, USA) and pSTAT3^ser727^ (#9134, Cell signaling, Danvers, MA, USA) in cohort 1. Staining for pSTAT3^tyr705^ was subsequently performed in cohort 2 and cohort 3 as validation. Briefly, sections were dewaxed and rehydrated through a series of graded alcohols. Antigen retrieval was performed using TRIS–EDTA pH9 (pSTAT3^tyr705^, pSTAT3^ser727^) or citrate buffer pH6 (JAK1, JAK2, STAT3) by heating for 5 min under pressure. Endogenous peroxidases were blocked in 3% H_2_O_2_ for 10 min. Sections were rinsed in water and then blocked using 5% casein (JAK1), 10% casein (STAT3) or 5% horse serum (pSTAT3^tyr705^) at room temperature. Primary antibodies were added (JAK1 (1:100), JAK2 (1:100), STAT3 (1:300), pSTAT3^tyr705^ (1:50), pSTAT3^ser727^ (1:400) and incubated overnight at 4 °C. Sections were washed in tris-buffered saline (TBS) and incubated for 30 min in ImmPRESS (Vector Laboratories Inc, Burlingame, CA, USA) (JAK1, JAK2, STAT3, pSTAT3^ser727^) or Envision (Agilent Technologies, Santa Clara, CA, USA) secondary (pSTAT3^tyr705^). Sections were washed again in TBS, and DAB substrate was added for 5 min. After rinsing in water, sections were counterstained in Harris haematoxylin, dipped in acid alcohol, blued in Scots tap water, dehydrated through a series of alcohols, and placed in Histoclear. Mounting was performed using Omnimount (HS-110, SLS, Nottingham, UK). Scanning was performed using a Hamamatsu Nanozoomer (Hamamatsu, Hertfordshire, UK) at X20 and images were visualized on the NDP Platform (Hamamatsu, Hertfordshire, UK).

The weighted histoscore method was utilised to semi-quantitatively measure the intensity of staining detected in the tumor cell nuclei (pSTAT3^tyr705^, pSTAT3^ser727^ and JAK1, JAK2) and expression in any part of stromal cells (pSTAT3^tyr705^). The proportion of nuclei were assessed for negative, weak, moderate, and strong staining for each marker by a single observer (KP) blinded to clinical outcomes. The following calculation allowed for a score ranging from 0–300 to be determined for every core; (0* % negative) + (1* %weak) + (2*%moderate) + (3*%strong). The TMA included 3 cores per patient to account for tumor heterogeneity. For validation of manual scores 10% of cores were scored digitally using QuPath [19] by a second observer (SAB) blinded to clinical outcomes. Scatter plots were constructed to visualize correlation between manual and digital scores and for each marker intra-class correlation coefficients of > 0.7 were achieved (Additional file 1, Figure S^1^).

### Immunohistochemical assessment of, pSTAT3^tyr705^ in AKPT and KPN murine tumors

Immunohistochemical staining to detect pSTAT3^tyr705^ was performed on a KPN and an AKPT tumor to determine constitutive activation of the pathway in vivo. A KPN male mouse on a C57BL/6J (generation N5-6) background of 10 weeks of age were induced with a single intraperitoneal injection of 2mg tamoxifen and aged until clinical endpoint as evidenced by anemia, hunching and/or weight loss to generate small intestinal tumor for IHC staining. Intracolonic induction in a 16-week-old female mouse *villin*Cre^ER^ *Apc*^fl/+^ *Kras*^G12D/+ ^*Trp53*^fl/fl ^*Tgfbr1*^fl/fl^ on a C57BL/6 background (generation N6/N7) was performed under general anesthesia. Three 70µl 100uM dose of 4-hydroxy tamoxifen (H7904-5MG from Sigma) were injected from the mid-colon to distal colon into the sub-mucosa via a colonoscope (Karl Storz TELE PACK VET X LED endoscopic video unit). At clinical endpoint (weight loss with the presentation of hunching) colonic tumor tissue (spontaneous SI tumour) was collected from a single tumour for IHC staining.

Staining was performed using pSTAT3^tyr705^ antibody (#9131, Cell signaling, Danvers, MA, USA) at 1:100 on a Leica BOND Rx autostainer (Leica Biosystems, Wetzlar, Germany).

### Assessment of pSTAT3^tyr705^ and pSTAT3^ser727^ colocalization in each compartment of the tumor microenvironment

Multiplex immunofluorescence was performed to determine the importance of colocalization of both phosphorylation sites of STAT3 in patients from cohort 1. TMAs were baked for 1 h at 60 °C. Dewaxing was performed using a PT module (Epredia, Runcorn, UK) by heating slides to 95 °C for 35 min in pH6 buffer (TA-999-DHBL, Dewax and HIER Buffer L, Epredia, Runcorn, UK). Staining was performed using a Lab Vision 480S Autostainer (Thermo Fisher Scientific, Waltham, MA, USA). Staining was performed using an UltraVision Quanto Detection System HRP kit (TL-060-QHL, Thermo Fisher Scientific, Waltham, MA, USA). Slides were washed in dH_2_O, quenched in 3% H_2_O_2_, washed in TBS and blocked in UVQ protein block (Thermo Fisher Scientific, Waltham, MA, USA). Sections were incubated with pSTAT3^tyr705^ (Cell signaling #9131) for 60 min and washed in TBS. UVQ amplifier (Thermo Fisher Scientific, Waltham, MA, USA) was applied for 10 min, washed off with TBS and then UVQ HRP (Thermo Fisher Scientific, Waltham, MA, USA) was added for 10 min. Sections were washed in TBS and incubated in Opal 480 at 1:300 (#SKU FP1500001KT, Akoya Biosciences, Marlborough, MA, USA) for 10 min before heating in the PT module. Slides were washed in dH2O, blocked in H_2_O_2_, washed in TBS, and blocked in UVQ protein block. Sections were incubated in pSTAT3^ser727^ antibody (Thermo Fisher Scientific, Waltham, MA, USA) at 1:400 for 30 min and then washed in TBST. UVQ amplifier was applied followed by washing in TBS, incubation with UVQ HRP, washing and addition of Opal 650 (SKU FP1496001KT, Akoya Biosciences, Marlborough, MA, USA) at 1:300 for 10 min. Sections were heated in the PT module for a final time, washed, incubated in DAPI for 5 min and then mounted using. Slides were scanned onto NDP using the Hamamatsu Nanozoomer.

### Assessment of the mutational profile of TSP^high^ patients with high and low pSTAT3 expression

Mutational profiling was performed on a subset of cohort 1 patients (*n* = 252). DNA was extracted from formalin fixed paraffin embedded sections by NHS Tayside Centre for Genomic Analysis (NHS Tayside, Dundee, UK). DNA quality and concentration were determined using the Qubit assay (ThermoFisher, Massachusetts, USA). Sequencing was outsourced and performed by Glasgow Precision Oncology Laboratory (GPOL) using a custom panel of 196 cancer-associated genes (Additional file 6, Table S^2^).

### Assessment of the bulk transcriptional profile of TSP^high^ patients with high and low pSTAT3 expression

Full CRC tissue resections from 49 patients from cohort 1 were profiled using TempO-Seq for detection of expression of the full transcriptome (~ 22,000 genes) (Biospyder Technologies, Carlsbad, CA, USA). This was performed as previously described [20]. Normalisation of raw gene counts was performed in R Studio (RStudio, Boston, MA, USA) using DESeq2. The expression profile of high TSP/pSTAT3^tyr705^ groups was analysed using publicly available software accessed at (https://www.gsea-msigdb.org/gsea/msigdb/index.jsp). Accession numbers for the dataset are provided within the data availability statement of the manuscript.

### NanoString digital spatial profiling (GeoMx™) of TSP^high^ patients with high pSTAT3 expression

GeoMx™ profiling was performed on a subset of CRC patients from cohort 2 (*n* = 11) as previously described. A tissue microarray from cohort 3 was cut at 5 μm and baked for 30 min at 60 °C. Antigen retrieval was performed using a Leica BOND Rx autostainer (Leica Biosystems, Wetzlar, Germany) using ER2 buffer at pH9 at 100 °C for 10 min. Protein digestion was performed using proteinase K (0.1 μg/ml) for 15 min. In-situ hybridisation of RNA-directed DNA oligo probes (Nanostring Human Whole Transcriptome Atlas, 18,677 genes) was performed according to the manufacturer’s protocol.

### Bioinformatics

Analyses to determine *STAT3* expression across publicly available datasets was performed using the confoundR application accessed at (https://confoundr.qub.ac.uk/) [21].

### Statistical analyses

Statistical analyses of IHC data were performed in SPSS version 28 (IBM, NY, USA). Kaplan Meier survival curves were plotted to determine association between groups and cancer-specific survival. To determine associations with clinicopathological features chi-squared tests were performed. Significance was set to *p* < 0.05. Cut points for high and low expression were determined in R Studio v1.4 (RStudio, MA, USA) R build version 4.2.1 and packages *Survminer* (version 0.4.9), *Maxstat* (version 0.7–25), *Tidyverse* (version 1.3.2) and *Survival* (version 3.5–0). These analyses determined the optimal cut off point using log rank statistics based on cancer-specific survival for cohort 1 and cohort 3, and disease-free survival for the TransSCOT clinical trial cohort (cohort 3).

Mutational profiling data were analysed in R Studio v1.4 (RStudio, MA, USA) using the *Maftools* (version 2.18.0) package. Fishers’ exact tests were utilized to determine any differential patterns of mutation between pSTAT3^tyr705^ and GMS patient groupings.

Normalized counts from the GeoMx experiment were downloaded and analysed in RStudio (v2022.07.2) using R build version 4.2.1. Differential Gene Expression (DGE) was performed using the Exact Test as part of edgeR package. Volcano plots were generated using *EnhancedVolcano*. Heat maps were generated using *ComplexHeatMap*. Gene set enrichment analysis (GSEA) was performed using the *fgsea* package. Single Sample Gene Set Enrichment Analysis (ssGSEA) was performed using the *GSVA* package. *ClusterProfiler* was used to interrogate the Reactome curated database. Immune spatial deconvolution of GeoMx derived WTA data was performed using *SpatialDecon* package. Receptor Ligand gene pairs were obtained from the CellPhoneDB repository.

Data from cell viability assays were analysed using paired T tests in GraphPad Prism version 9 (GraphPad Software, San Diego, CA, USA). Raw optical density read outs were averaged and normalized to vehicle controls. Paired t tests were used to determine any significant differences between treatment groups. To establish differences in immune profiles of high and low pSTAT3 groups within TSP^high^ cases Mann–Whitney unpaired non-parametric T tests were performed in GraphPad Prism version 9 (GraphPad Software, San Diego, CA, USA). Significance was set to *p* < 0.05.

All figures were constructed in Adobe Acrobat version 20 (Adobe Inc, San Jose, CA, USA).

## RESULTS

### JAK inhibitors reduced cell viability and proliferation of in vitro and ex vivo colorectal cancer models

**Fig. 1:**
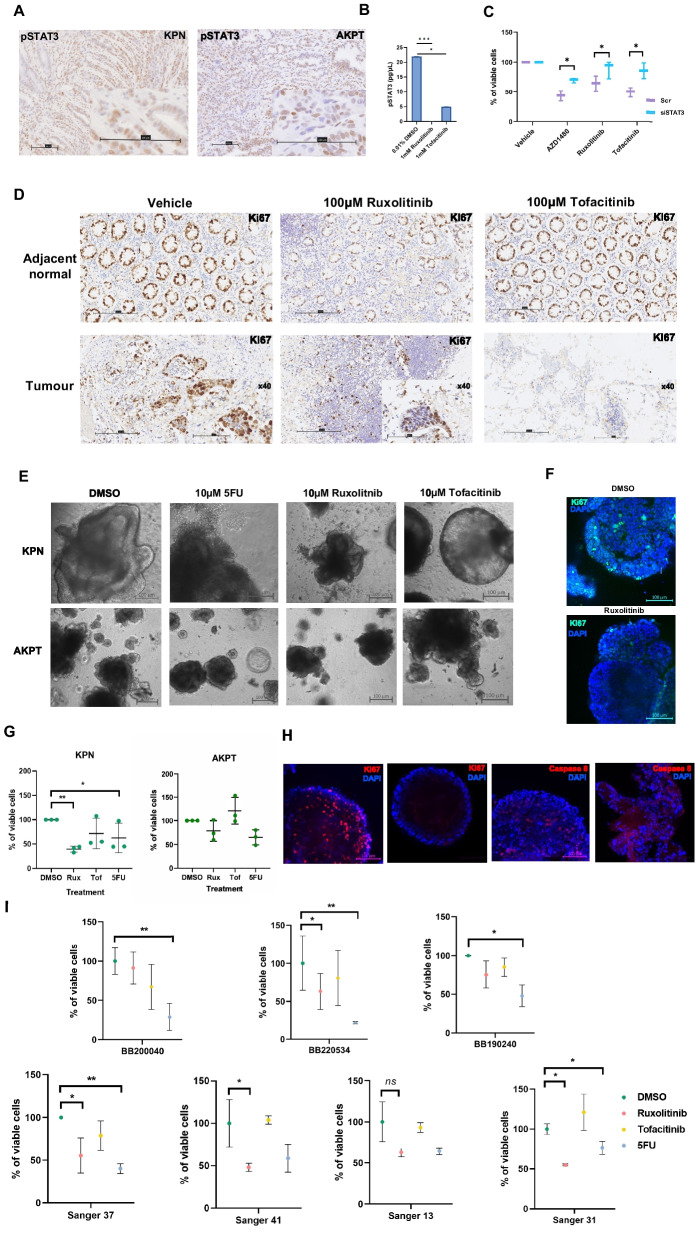
JAK inhibition and a therapeutic approach for CRC. Representative image of IHC staining for pSTAT3^tyr705^ in a KPN and AKPT mouse tumor (**A**). Bar chart showing the effect of JAK inhibitors on expression of pSTAT3^tyr705^ as measured by ELISA (**B**). Plot showing the effect of JAK inhibitors in control and in HCT116s with STAT3 knocked down (**C**). Representative images showing patient derived explants derived from matched normal and tumor colon samples and stained for Ki67via IHC after treatment with vehicle, Ruxolitinib or Tofacitinib (**D**). Brightfield images showing the effect of vehicle control, Ruxolitinib, Tofacitinib and 5FU on KPN and AKPT organoids after 72 h treatment (**E**). Representative images of immunofluorescent (IF) staining for Ki67 in an untreated and Ruxolitinib treated KPN mouse organoid (**F**). Box plot showing the effect of treatment with vehicle control, Ruxolitinib, Tofacitinib and 5FU for 72 h on cell viability of KPN and AKPT organoids (**G**). Representative images of IF staining for Ki67 and Caspase 8 in an untreated and Ruxolitinib treated PDO line (**H**). Dot plots showing the effect of treatment with vehicle control, Ruxolitinib, Tofacitinib and 5FU for 72 h on cell viability of 7 PDO lines with error bars represented by standard deviation and drug treatment compared to vehicle control by ANOVA (I). Significance set to *p < 0.05, **p < *0.005, p* < 0.0005***

Given that JAK inhibitors are already in use for myeloproliferative disorders and autoimmune diseases, we aimed to assess the potential for repurposing for CRC using in vitro and ex vivo models. Organoids derived from KPN (*VillinCre*^*ER−*^* Kras*^*G12D/*+^; *Trp53*^*fl/fl*^; *Notch1*^*TG*/+^) and AKPT (*VillinCre*^*ER*^*; Apc*^*fl/*+^*; Kras*^*G12D/*+^*; Trp53*^*fl/fl*^*;TgfbrI*^*fl/fl*^) were utilised to determine the effect of repurposed JAK1/2 inhibitor Ruxolitinib and JAK2/3 inhibitor Tofacitinib due to the high constitutive expression of pSTAT3 within tumor cell nuclei and CMS4 tumor classification (Fig. 1A). Both Ruxolitinib (*p* < 0.001) and Tofacitinib reduced pSTAT3^tyr705^ expression in HCT116 CRC cell lines when measured by ELISA (Fig. 1B). This was also observed in HT29 and DLD1 cells (Additional file 7 Fig. S^3^C). To test potential off-target effects of JAK inhibitors, HCT116 cells were silenced for STAT3 (Additional file 6, Fig. S^2^A, B), and cell viability of JAKi-treated cells were compared to controls. Cells with STAT3 knocked down had significantly higher cell viability compared to scramble control after treatment with AZD1480 (JAK2 inhibitor) (*p* = 0.032), Ruxolitinib (JAK1/2 inhibitor) (*p* = 0.011) and Tofacitinib (JAK2/3 inhibitor) (*p* = 0.029) (Fig. 1C). Patient derived explants were treated with Ruxolitinib and Tofacitinib to determine effects on matched normal and tumor tissue. Brightfield images show high Ki67 expression in normal tissue treated with vehicle and Tofacitinib, and moderate Ki67 in Ruxolitinib-treated explants (Fig. 1D). In contrast, tumor explants showed strong Ki67 staining and reduced expression in tumor explants treated with either inhibitor (Fig. 1D). Brightfield images show KPN and AKPT organoids 72 h after treatment with vehicle control, Ruxolitinib, Tofacitinib, and standard of care chemotherapeutic reagent 5-fluouracil (5FU) (Fig. 1E). KPN organoids showed decreased expression of Ki67 proliferation 72 h after treatment with Ruxolitinib (Fig. 1F). Cell viability was significantly reduced in KPN organoids treated with 10µM Ruxolitinib (*p* = 0.004) compared to control at 72 h and no significant response was observed in AKPT organoids (Fig. 1G).

When Ruxolitinib was tested in a panel of CRC cell lines, viability was significantly reduced in HCT116 at 10µM (*p* = 0.017) and 100µM (*p* = 0.01) and 1mM (*p* = 0.009). Similarly, cell viability was reduced at 1mM in HT29 (*p* = 0.0009), SW620 (*p* = 0.0002) and T84 (*p* = 0.005) (Additional file 3, Fig. S^3^A). Tofacitinib treatment significantly reduced viability of HCT116 cells at 10µM (*p* = 0.018) and HT29 at 1mM (*p* = 0.02) at 72 h (Additional file 3, Fig. S^3^B). Next the inhibitors were treated in patient-derived organoids. Representative immunofluorescent images show decreased expression of Ki67 and increased expression of Caspase-8 in a PDO line treated with 10µM Ruxolitinib (Fig. 1H). When a panel of PDOs were assessed for drug response 4/7 had significantly reduced cell viability 72 h after treatment with Ruxolitinib, compared to 5/7 with a significant response to 5FU when compared to vehicle control (Fig. 1H). None of the PDOs tested showed a response to Tofacitinib (Fig. 1I).

### JAK1 and JAK2 expression predict poor prognosis in CRC patients with stromal-rich tumors

**Fig. 2:**
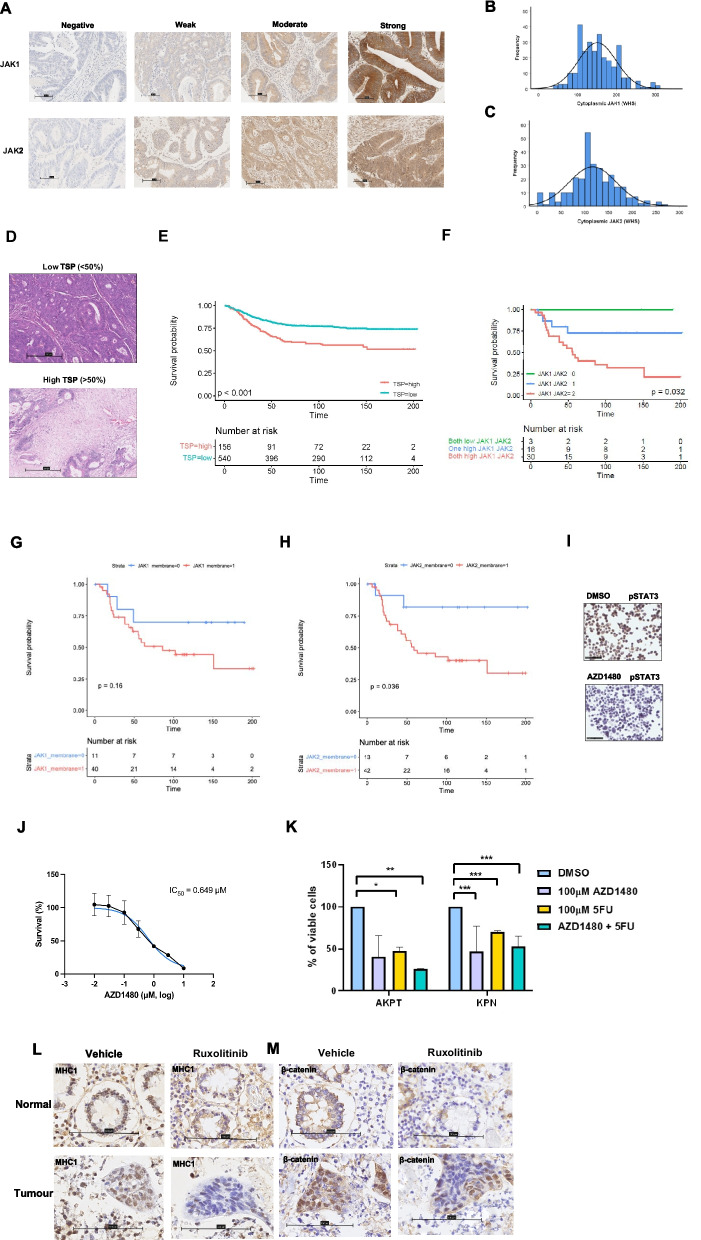
Expression of JAK1 and JAK2 and their association with prognosis in colorectal cancer. Representative images of negative, weak, moderate and strong IHC staining for JAK1 and JAK2 proteins in a retrospective cohort of CRC patients (**A**). Histograms showing the distribution of weighted histoscores for cytoplasmic JAK1 (**B**) and JAK2 (**C**). Representative images of high and low tumor stromal percentage cases (**D**). Kaplan Meier survival curve showing the association between TSP and CSS in cohort 1 (**E**). Kaplan Meier survival analysis of JAK1 and JAK2 tumor membrane expression in TSP^high^ cases in cohort 1 (**F**). Kaplan Meier survival analysis of membranous JAK1 and JAK2 expression in TSP^high^ cases when each protein was assessed individually (**G**-**H**). Representative staining of HCT116 cell pellets stained for pSTAT3^tyr705^ via IHC after treatment with vehicle of AZD1480 (**I**). Dose response curve showing the effect of JAK specific inhibitor AZD1480 on HCT116 cell viability (**H**). Bar chart showing the effect of 100µM AZD1480 on cell viability of mouse model organoids when used as monotherapy in AKPT (*p* = 0.056) and KPN (*p* < 0.0001) and in combination with 100µM 5FU (AKPT *p* = 0.002, KPN *p* = 0.0006) (**I**). Significance set to* p* < 0.05*, p < 0.005**, p < 0.0005***

Given the higher potency of Ruxolitnib (Jak1/2 inhibitor) over Tofacitinib (Jak2/3 inhibitor) in vitro and ex vivo, JAK1 and JAK2 protein expression was investigated in a large retrospective cohort of CRC patient tissue (cohort 1) (*n* = 1030) (Additional Fig. S^4^A). Positive staining for JAK1 and JAK2 was observed in the tumor cell cytoplasm and membranes of a subset of patients as shown in representative images of negative, weak, moderate, and strong staining shown in Fig. 2A. Staining quantification of cytoplasmic JAK1/2 showed normal distribution of weighted histoscores, and membrane staining was scored as absent or present (Fig. 2B-C). As previously published, TSP was assessed using routine diagnostic hematoxylin and eosin-stained resections (Fig. 2D), and high TSP (TSP^high^) was associated with significantly reduced cancer-specific survival (CSS) in cohort 1 (*p* < 0.001) (Fig. 2E). When scores for membranous JAK1 and JAK2 protein were assessed in cohort 1, high expression of both markers within TSP^high^ cases was associated with significantly reduced CSS (*p* = 0.032) (Fig. 2F). When membranous JAK1 and JAK2 expression was assessed individually within TSP^high^ cases, JAK1 was not associated with prognosis (*p* = 0.16) and high JAK2 expression was significantly associated with poor prognosis (*p* = 0.036) (Fig. 2G-H). Therefore, a preclinical JAK2 selective inhibitor, AZD1480 was tested in vitro. HCT116 cells treated with 10µM AZD1480 expressed less pSTAT3^tyr705^ than vehicle control proving JAK2 inhibition alone was sufficient to abrogate JAK/STAT3 signaling (Fig. 2H). AZD1480 treatment caused a significant reduction in cell viability of HCT116 CRC cell lines as shown by dose response curve in Fig. 2I AZD1480 (at 100µM) significantly reduced viability of both AKPT (*p* = 0.002) and KPN (*p* = 0.0006) when treated in combination with 100µM 5FU (Fig. 2J). In patient-derived explants, treatment with Ruxolitinib reduced expression of MHC1 and β-catenin in tumor samples, which suggests JAK inhibition may influence adaptive immunity and Wnt signalling (Fig. 2K). Further work is required to assess the clinical relevance of the dose of AZD1480 necessary to induce a response.

### Downstream of JAKs, STAT3 represents a prognostic marker and therapeutic target in CRC patients with stromal-rich tumors

**Fig. 3:**
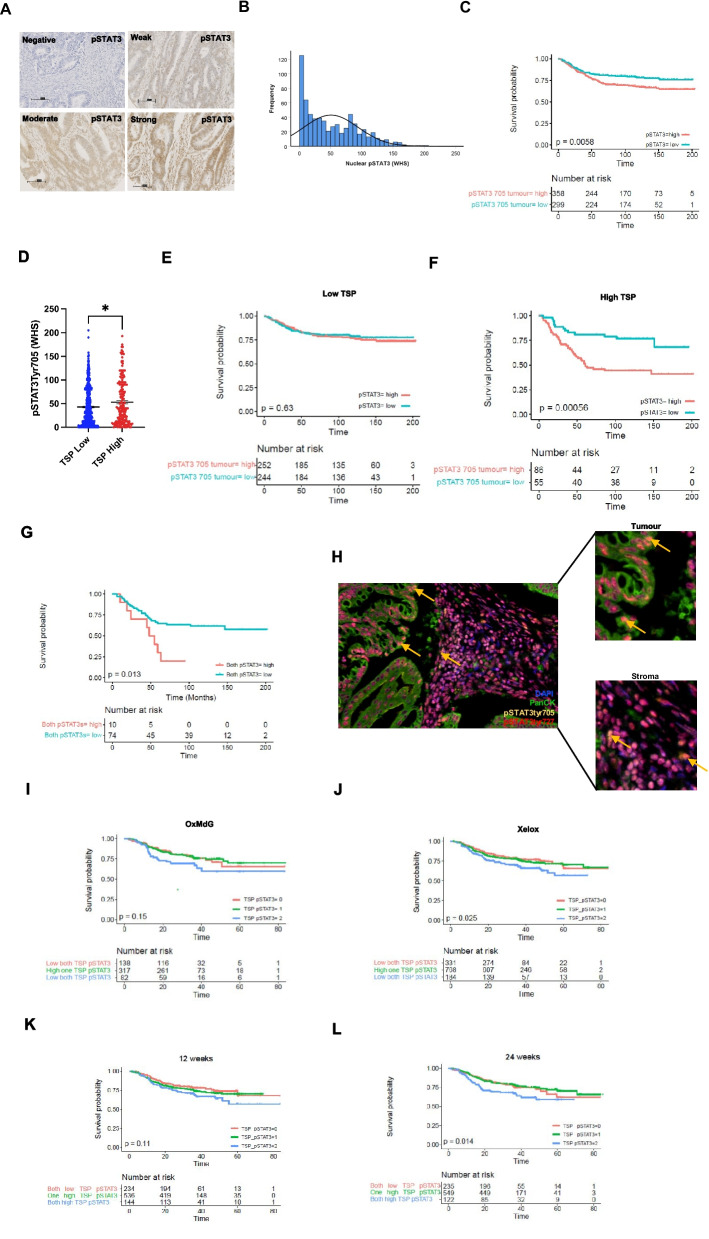
STAT3 expression is significantly prognostic in patients with stromal-rich tumors. Representative images of negative, weak, moderate, and strong IHC staining for pSTAT3^tyr705^ in CRC tissue from patient cohort 1 (**A**). Histogram showing the distribution of weighted histoscores for pSTAT3^tyr705^ quantified in the tumor cell nuceli of cohort 1 (**B**). Kaplan Meier curve showing the association between tumor nuclear expression of pSTAT3^tyr705^ and CSS in the full patient cohort 1(*n* = 660) (**C**). Bar plot showing the distribution of weighted histoscores for pSTAT3^tyr705^ across TSP^low^ and TSP^high^ cases (**D**). Kaplan Meier survival analysis showing tumor nuclear pSTAT3^tyr705^ expression and CSS in TSP^low^ (**E**) and TSP^high^ cases (**F**). Kaplan Meier survival curve showing the association between a combined score of pSTAT3^tyr705^ and pSTAT3^ser727^ expression and CSS in TSP^high^ cases (**G**). Representative image of multiplex IF staining for pSTAT3^tyr705^ and pSTAT3^ser727^ in CRC tissue from patient cohort 1 with arrows highlighting the dual positive cells (**H**). Kaplan Meier curves showing the association between pSTAT3^tyr705^ expression and CSS in the TransSCOT clinical trial patient cohort (*n* = 1820) relative to treatment type (**I**-**J**) and treatment duration (**K**-**L**). Significance set to* p* < 0.05*, p < 0.005**, p < 0.0005***

Upon ligand receptor binding of IL6/IL6R, JAK proteins become activated, which causes downstream phosphorylation of STAT3 at tyrosine 705 (pSTAT3^tyr705^). High expression of pSTAT3^tyr705^ can be detected in tumor cell nuclei of CRC tissues. Representative images of negative, weak, moderate, and strong staining of pSTAT3^tyr705^ are shown in Fig. 3A. When staining intensity was quantified in cohort 1 by weighted histoscore data showed a positively skewed distribution (Fig. 3B). In cohort 1 a cut point of 33.75 for high and low expression was determined using *Survminer* and high expression of pSTAT3^tyr705^ within tumor cell nuclei was detected in 54% of cases. Upon Kaplan Meier analysis high pSTAT3^tyr705^ was associated with reduced CSS (*p* = 0.006) (Fig. 3C). A consort diagram highlighting the number of patients analysed after exclusion criteria were applied is shown in Additional Fig. S^4^A. Expression of pSTAT3^tyr705^ was higher in TSP^high^ cases (*p* = 0.008) and there was potentiation of the survival effect of high pSTAT3^tyr705^ expression in TSP^high^ cases (*p* = 0.0006) (Fig. 3D-F). The association between pSTAT3^tyr705^ expression and clinical features is shown in (Additional file 6, Table S^2^).

Although pSTAT3^tyr705^ is the main marker of pathway activation, STAT3 can also be phosphorylated at pSTAT3^ser727^, therefore expression of pSTAT3^ser727^ was assessed in cohort 1. A high combined score of pSTAT3^tyr705^ and pSTAT3^ser727^ significantly associated with reduced CSS in TSP^high^ cases (*p* = 0.0013) (Fig. 3G). IF staining was performed to assess whether these 2 markers colocalize and positive staining was detected in some tumor cells as shown by arrows highlighting these dual positive cells (Fig. 3H).

Next the expression of pSTAT3^tyr705^ was assessed in a large and unique prospectively collected clinical trial cohort, TransSCOT (*n* = 2900) which explored whether different durations of 2 types of chemotherapy were inferior or superior. The number of patients included in the final analyses are shown in a consort diagram in (Additional file 4, Fig. S^4^C). Patients classified as high for both pSTAT3^tyr705^ and TSP^high^ had reduced cancer-specific survival when treated with Xelox (*p* = 0.025) (Oxaliplatin and capecitabine (CAPOX))) but not OxMdG (Folinic acid, fluorouracil and oxaliplatin (FOLFOX)) (*p* = 0.15) (Fig. 3I-J). Patients classified as high for both pSTAT3^tyr705^ and TSP^high^ also observed significantly reduced survival times when treated for 24 weeks (*p* = 0.014) but not 12 weeks duration (*p* = 0.11) (Fig. 3K-L). These data suggest that JAK inhibitors could be of benefit in patients receiving Xelox therapy.

### High pSTAT3 within stromal-rich tumors is underpinned by distinct immune profiles, mutational landscape and patterns of gene expression

**Fig. 4:**
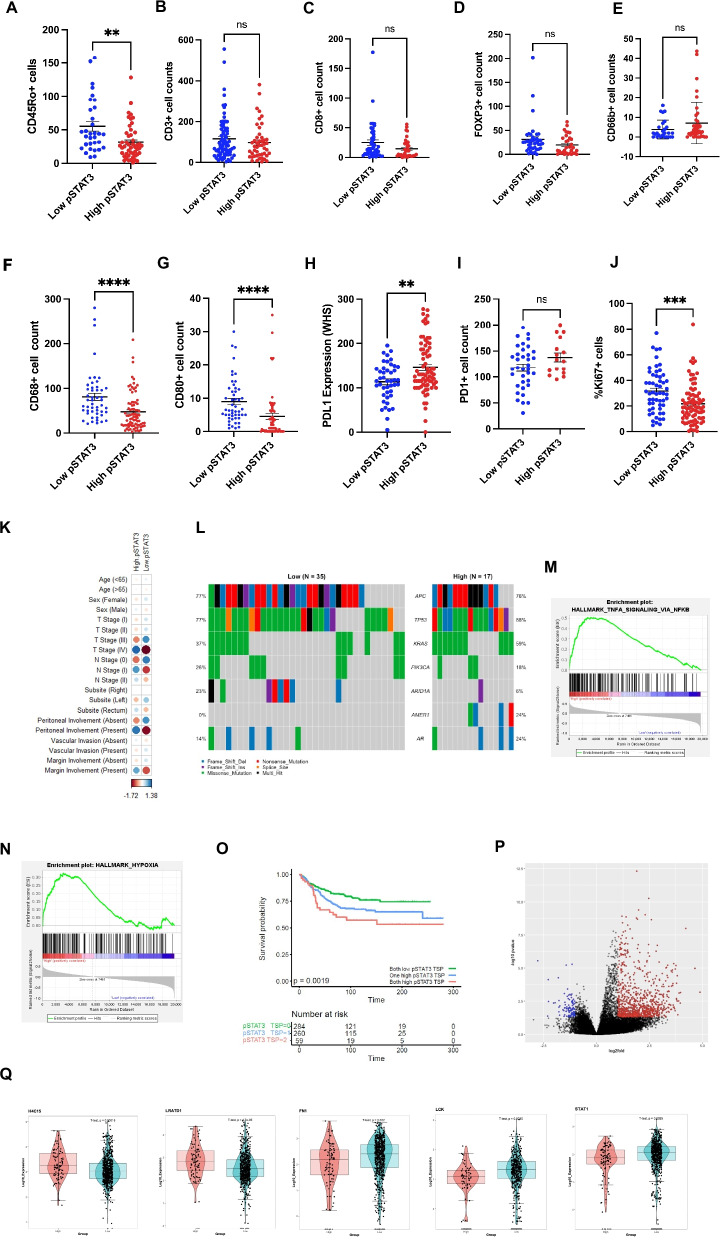
Immune and genetic profiles of high pSTAT3 expression within TSP^high^ cases. Box plot showing association between CD45Ro + (**A**), CD3 + (**B**), CD8 + (**C**), FOXP3 + (**D**), CD66b + (**E**), CD68 + (**F**), CD80 + (**G**), PDL1 + (**H**), PD1 + (**I**) and Ki67 + cells (**J**) and pSTAT3^tyr705^ status within TSP^high^ tumors. Statistical significance was assessed using Mann–Whitney tests and bars represent mean ± standard error of the mean. Corrplot showing the association between pSTAT3 status and clinicopathological characteristics of TSP^high^ patients (**K**). Oncoplot showing the top mutated genes between high and low pSTAT3^tyr705^ groups within the TSP^high^ patients (**L**). Enrichment plots showing the key pathways upregulated in pSTAT3^tyr705^ high TSP^high^ tumors (**M**–**N**). Kaplan Meier survival curve showing the association between pSTAT3^tyr705^ and TSP score in cohort 3 (*p* = 0.0019) (**O**). Volcano plot showing differential gene expression analysis of high versus low pSTAT3^tyr705^ within TSP^high^ cases (**P**). Box plots showing top upregulated and downregulated genes across pSTAT3^tyr705^ high and low groups within TSP^high^ tumors of cohort 3 (**Q**). Significance set to* p* < 0.05*, p < 0.005**, p < 0.0005***, p < 0.00005****

Next, the additional phenotypes associated with high STAT3^tyr705^ expression in TSP^high^ cases was explored in cohort 1. Immune populations in the tumor stroma were assessed for associations relative to tumoral pSTAT3^tyr705^ within the TSP^high^ tumors. Immune counts were performed manually from IHC staining of the TMA by a single observer blinded to clinical outcome (JI) with 10% of cases validated by a second observer (AR). Cells positive for each marker within the tumor-associated stroma of each TMA core were counted at total magnification of X400 and the scores for cases in triplicate averaged. The number of CD45Ro + cells detected in the tumor-associated stroma was lower in the high pSTAT3 cases (*p* = 0.002) (Fig. 4A). There was no significant difference in overall T cell infiltration (*p* = 0.3310), cytotoxic T cells (*p* = 0.078) or regulatory T cells (*p* = 0.084) between pSTAT3 high and low tumors (Fig. 4B-D). Similarly, no difference in granulocyte infiltration was observed (*p* = 0.174) (Fig. 4E). A significant reduction in macrophages was observed in high pSTAT3 tumors (*p* = 0.0002) and CD80 + cells (M1-like) (*p* = 0.0011) (Fig. 4F-G). There were significantly increased cells expressing checkpoint protein PDL1 (*p* = 0.0087) but not PD1 (*p* = 0.1744) (Fig. 4H-I). The % of Ki67 + tumor cells was significantly lower in tumors with high pSTAT3 (*p* = 0.0002) (Fig. 4J).

Chi-squared tests revealed a significant association between high tumoural pSTAT3^tyr705^ and T stage (*p* = 0.034), age (*p* = 0.012) and peritoneal involvement (spread to the peritoneum) (*p* = 0.012) in TSP^high^ cases in cohort 1 (Fig. 4K, Additional file 6, Table S^2^). Panel mutational profiling was available for a subset of these patients, which showed a significant enrichment for regulator of Wnt signaling pathway *APC recruitment protein-1* (*AMER1)* mutation in pSTAT3^tyr705^ high TSP^high^ cases as shown in an oncoplot (Fig. 4L). *AMER1* is a regulator of the Wnt signaling pathway. Similarly, bulk *RNA* sequencing data were available for a subset of patients, and this showed that high pSTAT3^tyr705^ within TSP^high^ cases was enriched by Hallmark pathways including *TNFα signaling *via* NFκB* (ES = 0.51, *p* = 0.001) and *Hypoxia* (ES = 0.32, *p* = 0.05) (Fig. 4M-N).

To elucidate association of high pSTAT3^tyr705^ in TSP^high^ with transcriptomic profiles expression was assessed in a 3rd retrospective cohort of CRC patients (cohort 3) with multiomic data available. The number of patients included in the final analyses are highlighted in a consort diagram in Additional file 3, Fig. S^4^. Assessment of the combination of pSTAT3^high^ and TSP^high^ was associated with significantly worse outcome (*p* < 0.001) (Fig. 4O) validating findings from cohorts 1 and 2. Differential gene expression analysis of bulk *RNA* sequencing data revealed differences between high and low pSTAT3^tyr705^ tumors in TSP^high^ tumors (Fig. 4P). Genes including histone *H4C15* (*p* < 0.001) and *LRATD1* (*p* < 0.001), which is involved in cell motility were upregulated in the pSTAT3 TSP^high^ cases (Fig. 4Q). Conversely, *Fibronectin-1* (*FN1)* (*p* = 0.022), Src family member *lymphocyte cell-specific protein-tyrosine kinase* (*LCK)* (*p* = 0.0035) and *signal transducer and activator of transcription-1* (*STAT1)* (*p* = 0.0025) were downregulated (Fig. 4Q).

### Spatial profiling of tumor, stroma and immune components of the microenvironment identified upregulation of cancer-promoting genes and pathways in high pSTAT3 high TSP cases

**Fig. 5:**
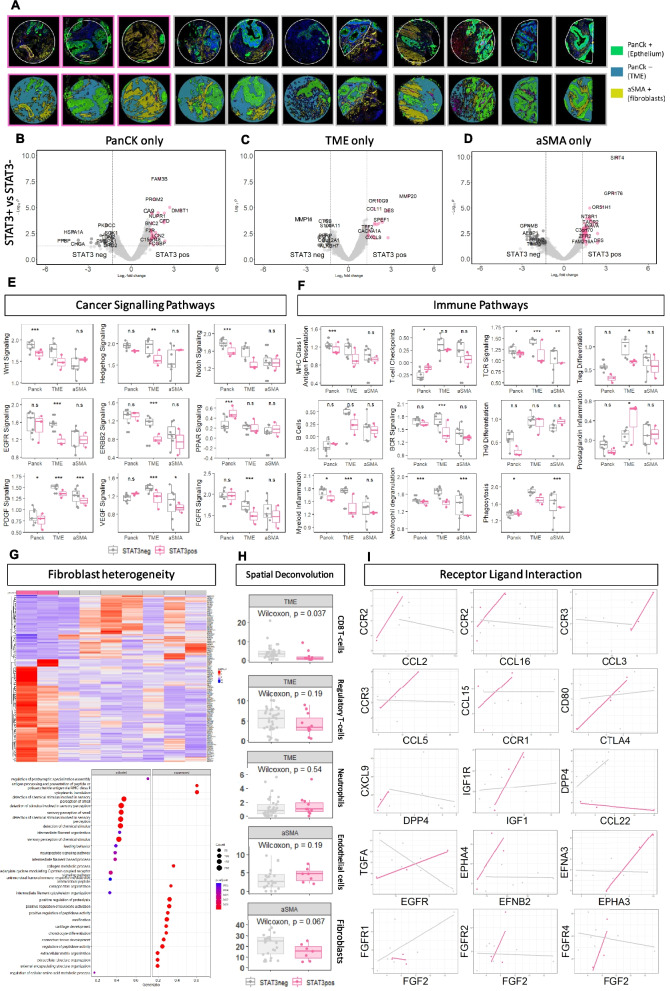
Digital Spatial Profiling revealed profound differences in compartments of the tumor microenvironment of high pSTAT3^Tyr705^ cases. First row demonstrates representative images of 3 high pSTAT3 TSP^high^ TMA cores (pink) and 8 Low pSTAT3 TSP^high^ TMA cores (from patient cohort 3) (grey) stained with SYTO13 (blue), PanCK(green), aSMA(yellow) scanned on the Nanostring GeoMx® platform (**A**). The second row demonstrates masks used to generate PanCK + (epithelial), aSMA + (fibroblast), PanCK-SMA-(TME) segmented transcriptome compartments (**A**). Volcano plot demonstrating differential gene expression results comparing high pSTAT3 cores versus low pSTAT3 cores in PanCK compartment only (**B**). Volcano plot demonstrating differential gene expression results comparing high pSTAT3 cores versus low pSTAT3 cores in TME compartment only (**C**). Volcano plot demonstrating differential gene expression results comparing high pSTAT3 cores versus low pSTAT3 cores in aSMA compartment only (**D**). Box plots demonstrating Normalized Expression (y-axis) of common cancer signaling pathways (Nanostring WTA curated gene sets) calculated using fgsea comparing high pSTAT3 (pink) and low pSTAT3 (grey) cases in segmented compartments (x-axis) (*p* < 0.05*,* p* < 0.005**, *p* < 0.0005***) (**E**). Box plots demonstrating Normalized Expression (y-axis) of common immune signaling pathways (Nanostring WTA curated gene sets) calculated using fgsea comparing high pSTAT3 (pink) and low pSTAT3 (low) cases in segmented compartments (x-axis) (*p* < 0.05*, *p* < 0.005**, *p* < 0.0005***) (**F**). Panel 1: Heatmap demonstrating differentially expressed genes comparing high pSTAT3 and low pSTAT3 in aSMA compartment. Panel 2: Gene Set Enrichment Analysis of differentially expressed genes in the aSMA compartment comparing high pSTAT3 and low pSTAT3 in aSMA compartment generated using ClusterProfiler package to interrogate REACTOME curated pathways (**G**). Boxplots demonstrating results of deconvoluting transcriptomic readout of high pSTAT3 and low pSTAT3 cores into estimated immune cells counts, selected plots from TME and aSMA compartment are shown (**H**). Scatterplots of gene expression count of selected receptor ligand pairs (CellPhoneDB) comparing high pSTAT3 (pink) and low pSTAT3 (grey) with superimposed linear model line, well correlated gene pairs are assumed to imply a relationship between receptor and ligand in that group (**I**)

To further cellular and molecular characteristics associated with high pSTAT3^tyr705^ within TSP^high^ phenotypes, Nanostring GeoMx® digital spatial profiling was performed on a subset of cohort 2 tumor samples using a TMA. TSP^low^ cases were excluded for this analysis. 3 patients classified as high pSTAT3 (pSTAT3^tyr705^ from IHC data) and TSP^high^ (high TSP from H&E) were identified, with representative images of TMA cores shown in Fig. 5A. There were 8 cases classified as low for either or both pSTAT3/TSP (Fig. 5A). Representative images of multiplex immunofluorescent (mIF) staining tumor epithelium (pan cytokeratin/panCK) and stroma (alpha-smooth muscle actin/αSMA) are shown in Fig. 5A. When panCK + regions were analysed, several genes were enriched in high STAT3 TSP^high^ cases including hypoxia-associated carbonic anhydrase-9 (*CA9*) (Fig. 5B). The TME (panCK negative and αSMA negative) in high pSTAT3^tyr705^ TSP^high^ cases was enriched for inflammation associated gene *CXCL9*, and eosinophil chemoattractant protein (*CCL11)* and matrix metallopeptidase 9 *(MMP9)* (Fig. 5C). CXCL9 plays a role in recruitment of T cells, CCL11 promotes eosinophil chemotaxis and MMP9 is involved in tissue remodelling. Stromal regions (αSMA +) of high pSTAT3^tyr705^/TSP^high^ cases had higher expression of tumor suppressor gene *SIRT4* and G-protein coupled receptor 176 (*GPR176)* (Fig. 5D).

When common cancer-associated signaling pathways were assessed, there was decreased expression of classical CRC-associated pathways *Wnt* and *Notch* signaling in panCK + tissue of high pSTAT3^tyr705^ TSP^high^ (Fig. 5E). In the TME (panCK- and αSMA-) there was lower expression of epidermal growth factor receptor (*EGFR)*, vascular endothelial growth factor (*VEGF)* and fibroblast growth factor receptor (*FGFR)* signaling pathways in high pSTAT3^tyr705^/TSP^high^ versus other cases (Fig. 5E). In αSMA + regions platelet-derived growth factor (*PDGF)* and *VEGF* signaling were enriched in the low STAT3 and TSP^high^ group (Fig. 5E). When immune related pathways were assessed in the panCK + tissue high pSTAT3^tyr705^ TSP^high^ had significantly reduced expression of MHC class I presentation and increased T cell checkpoints. (Fig. 5F). In the TME there was reduced T cell Receptor (TCR) signaling, reduced B cell receptor signaling and increased prostaglandin inflammation (Fig. 5F). In the αSMA + tissue, high pSTAT3^tyr705^ TSP^high^ had reduced TCR signaling, reduced Neutrophil degranulation and reduced phagocytosis (Fig. 5F).

Differential gene expression demonstrated a markedly different gene signature between pSTAT3 high panCK and αSMA- regions (Fig. 5G). Pathway analysis of this signature demonstrated loss of extracellular matrix organization suggesting a loss of normal fibroblast function in high pSTAT3^tyr705^ TSP^high^ (Fig. 5G). Spatial deconvolution of the TME showed a reduction in CD8 + T cells, regulatory T cells, fibroblasts and an increase in neutrophils and endothelial cells in high pSTAT3^tyr705^ TSP^high^ cases (Fig. 5H). Conversely, despite higher numbers of neutrophils in pSTAT3 + patients, there was a reduction in neutrophil degranulation suggesting a loss of normal neutrophil function. Receptor ligand interaction analysis showed increased likelihood of interaction between CCR2/CLL2, CCR2/CCL16, CCR3/CCL3, CCR3/CCL5, CCR1/CCL15, CTLA4/CD80, DPP4/CXCL8, IGF1R/IGF1, EPHA3/EFNA3 and FGFR2/FGF2 in the high pSTAT3 TSP^high^ cases (Fig. 5I). In the pSTAT3 low cases there was increased likelihood of interaction between DPP4/CCL22 and FGFR1/FGF2 (Fig. 5I).

In summary, spatial profiling of CRC tissue revealed differences in the gene expression profiles of high versus low pSTAT3^tyr705^ in TSP^high^ cases in the tumor cells themselves, the surrounding stromal cells, and the rest of the microenvironment. High pSTAT3 TSP^high^ cases demonstrated enrichment for reduced MHC-1 presentation coupled with reduced CD8 + cells within the tumor microenvironment highlighting impaired anti-tumor immunity. These tumors also demonstrated upregulation of genes associated with markers of hypoxia and stromal remodelling.

### STAT3 signaling the stroma is prognostic and can also be targeted using JAK inhibitors

**Fig. 6:**
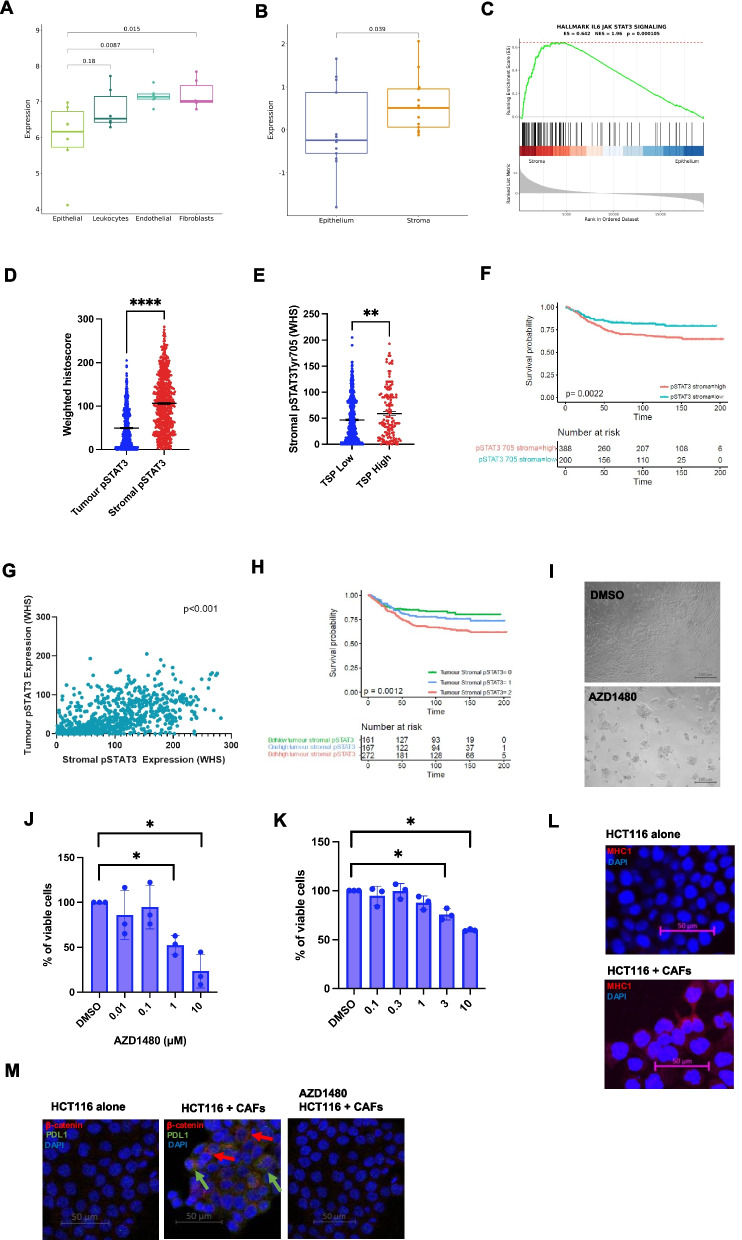
Activation of STAT3 in the tumor-associated stroma. Box plot showing the expression of STAT3 in different cell populations from a cohort of CRC patients from counfoundR (**A**-**B**). Enrichment plot showing IL6/JAK/STAT3 in a CRC cohort comparing epithelial and fibroblas t expression using confoundR (**C**). Box plot showing the expression of pSTAT3^tyr705^ within the tumor and stromal compartments of cohort 1 (**D**). Box plot showing the expression of pSTAT3^tyr705^ within the stroma relative to TSP status of cohort 1 (**E**). Kaplan Meier survival curve showing the association between stromal pSTAT3 and CSS in cohort 1 (**F**). Scatter plot showing the association between pSTAT3 expression within the tumor and stroma of cohort 1 (**G**). Kaplan Meier survival curve showing the association between a combined tumor and stromal score for pSTAT3^tyr705^ and CSS in cohort 1 (**H**). Brightfield images of untreated/AZD1480 treated CCD18Co colorectal fibroblasts (**I**). Bar chart showing the effect of AZD1480 on cell viability of CCD18Co cells (**J**) and a patient-derived CAF primary cell line (K). Representative images of IF staining for MHC1 in HCT116 cells grown as a monoculture and as a coculture with CAFs (**L**) Representative images of IF staining for β-catenin and programmed death ligand 1 (PDL1) in HCT116 cells grown as a monoculture, as a coculture with CAFs treated with vehicle control or AZD1480 (**M**). Significance set to* p* < 0.05*, p < 0.005**, p < 0.0005***, p < 0.00005****

Data from previous studies have shown the importance of JAK/STAT signaling not only in tumor cells themselves but also in the surrounding stroma. Analysis of publicly available CRC datasets using counfoundR (https://confoundr.qub.ac.uk/) showed that expression of *STAT3* is significantly higher in fibroblast populations compared to epithelial cells (*p* = 0.015) (Fig. 6A). Similarly in another CRC dataset expression of *STAT3* and the hallmark *IL6/JAK/STAT3* pathway was enriched in the stroma compared to epithelium (*p* = 0.039) (Figs. 6B-C). In cohort 1, the expression of pSTAT3^tyr705^ was higher within the stroma than the epithelium (*p* < 0.0001) (Fig. 6D). Stromal expression of pSTAT3^tyr705^ was higher in TSP^high^ cases in cohort 1 (*p* = 0.002) (Fig. 6E). Kaplan Meier survival analysis showed a significant association between high expression of stromal pSTAT3^tyr705^ and reduced CSS in cohort 1 (Fig. 6F). Multivariate cox regression analysis showed that stromal pSTAT3 expression was an independently prognostic factor (*HR* = *1.691, 95%CI; 1.123–2.547, p* = *0.012*) (Additional file 7, Table S^3^). There was a moderate but significant correlation between the expression of stromal and tumor pSTAT3^tyr705^ (*Rho* = *0.51, p* < *0.0001*) (Fig. 6G). When pSTAT3 stromal and tumor scores for cohort 1 were combined, Kaplan Meier survival analysis showed that high expression in both areas was associated with significantly worse CSS (*p* = 0.001) (Fig. 6H). JAK2 inhibitor AZD1480 reduced cell viability in a colon fibroblast cell line compared to vehicle as shown by brightfield images (Fig. 6I) and cell viability assay (*p* = 0.007) (Fig. 6J). AZD1480 also significantly reduced cell viability of patient-derived cancer-associated fibroblasts (CAFs) at 3 μM (*p* = 0.018) and 10 μM (*p* = 0.0002), however at 10 μM the % of viable cells was still > 50% (Fig. 6K). To validate data from the GeoMx experiments, HCT116 cells were grown as a coculture with CAFs and stained for MHC1. Representative images show increased MHC1 expression in tumor cells grown with CAFs versus as monocultures (Fig. 1L). Subsequently, HCT116 cells were grown in a coculture with CAFs and treated with either vehicle control or 10 μM AZD1480 and stained for PDL1 (checkpoint protein) and β-catenin (Wnt activity). Representative images of IF staining show HCT116 cells grown alone had low expression of PDL1 and β-catenin and adding in CAFs t the culture increased expression of both markers (Fig. 6M). Treatment with AZD1480 reduced the expression of PDL1 and β-catenin which implicates both stromal involvement and STAT3 in driving checkpoint and Wnt activity expression (Fig. 6M).

## Discussion

In this study a novel association between JAK/STAT3 signaling pathway activation and poor prognosis has been identified in a specific subset of patients with stromal-rich (TSP^high^) CRC tumors. Previous research has implicated STAT3 in CMS1 and CMS3 tumors [22]. However, here we have shown that high expression of the main marker of pathway activation pSTAT3^tyr705^ was associated with poor outcome in TSP^high^ cases, which more closely resemble CMS4 (*p* = 0.006).

This interaction between STAT3 signaling and tumor-stroma has been noted in previous literature. In mouse models of pancreatic ductal adenocarcinoma (PDAC), inhibiting STAT3 in the tumor resulted in stromal remodelling [23]. In vitro cell line studies of PDAC have shown that conditioned medium from Cancer Associated Fibroblasts (CAFs) induced STAT3 expression in PDAC-3 tumor cells at the transcriptomic and protein level [23–25]. Similarly in models of non-small cell lung cancer (NSCLC) culturing tumor cells with primary CAFs induces STAT3 in the tumor cells and induces EMT [25].

In the present study, JAK inhibition reduced cell viability expression pf pSTAT3^tyr705^ in HCT116 and HT29 CRC cell lines. Similarly, mouse derived KPN organoids and a subset of PDOs showed a significant decrease in the percentage of viable cells, apoptosis and proliferation following JAK/STAT3 inhibition using Ruxolitinib and AZD1480. In this study we showed JAK inhibitors were significantly less effective in cells silenced for STAT3, however off target effects should be investigated in future studies. Further work is needed to understand if this difference in responses and unravel the mechanisms underlying why specific PDOs showed better responses to JAKi.

Across 3 large CRC patient cohorts, high expression of pSTAT3^tyr705^ was associated with reduced CSS. This included data from the TransSCOT clinical trial cohort where we were able to show that patients with high pSTAT3^tyr705^ and TSP^high^ had significantly better responses to FOLFOX over CAPOX regimes, and to treatment for 12 weeks over 24 weeks duration. This result needs to be validated in a subsequent cohort, however, highlights the potential for improving outcomes to existing therapeutics in the pSTAT3 high TSP^high^ group.

To understand association with other phenotypes, mutational, bulk transcriptional, spatial, and immune profiling revealed differences in the tumors of patients with high pSTAT3 and TSP^high^. In the mutational analyses of patient cohort 1, there was an enrichment of *AMER1* in the high pSTAT3^tyr705^ group, but significantly less *SMAD4* mutations. These data suggested that aberrant JAK/STAT3 signaling in the CRC setting is unlikely to be mutationally driven. At the bulk transcriptomic level, differences were observed between patients with high/low pSTAT3^tyr705^ in patient cohort 1, however the biological relevance if this needs further investigation, due to overlap between groups despite statistical significance. Pro-tumorigenic Hallmark pathway *TNFA signaling *via* NF-κB*, and *Apoptosis* enriched in TSP^high^ patients classified as high pSTAT3^tyr705^. Digital spatial profiling of a subset of patients from patient cohort 3 enabled interrogation of differential gene expression within the panCK positive, αSMA positive and TME areas of high pSTAT3 TSP^high^ cases. These data have highlighted the milieu of dysregulated signaling in different spatial compartments of stromally dense STAT3 activated tumors. There was an upregulation of genes associated with immune exclusion, decreased anti-tumor immune response, altered neutrophil biology, non-classical fibroblast deposition and increased hypoxia in TSP^high^ tumors with high pSTAT3. This was in concordance with data from the protein level from cohort 1 which showed high tumoral expression of PDL1 in TSP^high^ tumors classified as high for pSTAT3^tyr705^. However, the biological relevance if this needs further investigation, due to overlap between groups despite statistical significance. Future research is required to validate these mechanisms in vitro*/*in vivo, and to determine if inhibition of STAT3 signaling can reverse the adverse phenotypes acquired in the high pSTAT3 TSP^high^ tumors.

While the effect of STAT3 activation in tumor cells on the inflammatory infiltrate is well characterized, the effects on the tumor-stroma is less well-studied [26]. The interplay between all 3 components of the TME is likely responsible for driving phenotypes which predict poor prognosis. We hypothesise that a feedback loop arises, whereby cytokines secreted by CAFs activate STAT3 in the tumor cells, which causes transcription of genes which promote stromal cell proliferation and recruitment of CD66b + cells to the TME. The increased stromal component is linked to tumor budding, hypoxia and EMT [4, 27–29]. Activation of STAT3 in tumor cells causes increased expression of PDL1 resulting in immune evasion [30]. Blocking STAT3 activation in patients with stromal-rich tumors could suppress these hallmarks of cancer and prevent tumor progression.

There is evidence that IL6 can not only activate STAT3 but also other STAT family members including STAT1. High expression of STAT1 generally confers good prognosis in solid tumors through promotion of anti-tumor immunity [31]. Indeed, in previous tissue-based studies the ratio of STAT1:STAT3 in tumor cells was highly prognostic in CRC, and in prostate cancer loss of STAT1 associated with increased recurrence [32, 33]. Further mechanistic work is needed to determine whether blocking STAT3 switches signal transduction to STAT1, in this study *STAT1* gene expression was upregulated in low STAT3 TSP^high^ tumors (*p* = 0.0025). Future work should include coculture models to investigate JAKi effect on tumor cells in the presence of CAFs and various immune cell populations.

In conclusion, patients with TSP^high^ tumors have the worst outcomes, regardless of segregation strategy including transcriptional level (CMS4/CRIS-B) or the histological level. This subgroup of patients warrants urgent identification of novel therapeutic options to improve survival. From this study, we have shown that JAK inhibitors should be investigated using a stratified medicine approach for patients with stromal-rich TSP^high^ tumors.

## Supplementary Information

**Additional file 1: Figure S1.** Validation of manual scoring using digital scoring.

**Additional file 2: Figure S2.** Validation of silencing STAT3 in HCT116.

**Additional file 3: Figure S3.** Assessment of JAK inhibitors in vitro.

**Additional file 4: Figure S4.** Patients included in analysis of retrospective cohorts.

**Additional file 5:** **Table S1.** List of genes included in the mutation panel.

**Additional file 6: Table S2.** Chi-squared table of association between pSTAT3tyr705 and clinical features.

**Additional file 7. Table S3.** Univariate and multivariate cox regression on cohort 1.

## Data Availability

Data are stored within the glasgow safehaven database (gsh21on009, gsh/18/on007). transcriptomics data are deposited with arrayexpress at e-mtab-1307 for cohort 1 and bioproject accession prjna997336 for cohort 2.

## Abbreviations

αSMA: Alpha smooth muscle actin
CAFs: Cancer-associated fibroblast
CAPOX: Oxaliplatin and capecitabine
CMS: Consensus molecular subtypes
CRC: Colorectal cancer
CSS: Cancer-specific survival
CRIS: Cancer cell intrinsic subtypes
EMT: Epithelial to mesenchymal transition
FOLFOX: Folinic acid, fluorouracil, and oxaliplatin
IL6: Interleukin 6
IL6R: Interleukin 6 receptor
JAK: Janus Kinase
MHC1: Major histocompatibility complex 1
NSCLC: Non-small cell lung cancer
PDL1: Programmed death ligand 1
PDO: Patient-derived organoid
STAT3: Signal transducer and activator of transcription 3
STAT1: Signal transducer and activator of transcription 1
TSP: Tumour stroma percentage
