# Correction: JAK/STAT3 represents a therapeutic target for colorectal cancer patients with stromal‑rich

**DOI:** 10.1186/s13046-026-03811-6

**Published:** 2026-08-15

**Authors:** Kathryn A. F. Pennel, Phimmada Hatthakarnkul, Colin S. Wood, Guang‑Yu Lian, Sara S. F. Al‑Badran, Jean A. Quinn, Assya Legrini, Jitwadee Inthagard, Peter G. Alexander, Hester van Wyk, Ahmad Kurniawan, Umar Hashmi, Michael A. Gillespie, Megan Mills, Aula Ammar, Jennifer Hay, Ditte Andersen, Colin Nixon, Selma Rebus, David K. Chang, Caroline Kelly, Andrea Harkin, Janet Graham, David Church, Ian Tomlinson, Mark Saunders, Tim Iveson, Tamsin R. M. Lannagan, Rene Jackstadt, Noori Maka, Paul G. Horgan, Campbell S. D. Roxburgh, Owen J. Sansom, Donald C. McMillan, Colin W. Steele, Nigel B. Jamieson, James H. Park, Antonia K. Roseweir, Joanne Edwards

**Affiliations:** 1 School of Cancer Sciences, Wolfson Wohl Cancer Research Centre, University of Glasgow, Glasgow, G61 1QH UK; 2 Department of Surgery, Glasgow Royal Infirmary, Glasgow, G31 2ER UK; 3 University of Glasgow Medical School, Glasgow, G12 8QQ UK; 4 CRUK Scotland Institute, Glasgow, G61 1BD UK; 5 Glasgow Tissue Research Facility, Queen Elizabeth University Hospital, Glasgow, G51 4TF UK; 6 Bioclavis Ltd, Queen Elizabeth University Hospital, Glasgow, G51 4TF UK; 7 CRUK Clinical Trials Unit, The Beatson West of Scotland Cancer Centre, Gartnavel Hospital, Glasgow, G12 0XH UK; 8 Wellcome Centre for Human Genetics, University of Oxford, Oxford, OX3 7BN UK; 9 NIHR Oxford Biomedical Research Centre, Oxford University Hospitals NHS Foundation Trust, John Radcliffe Hospital, Oxford, OX3 9DU UK; 10 Edinburgh Cancer Research Centre, IGMM, , University of Edinburgh, Crewe Road, Edinburgh, EH4 2XU UK; 11 The Christie NHS Foundation Trust, Manchester, M20 4BX UK; 12 Southampton University Hospital NHS Foundation Trust, Southampton, SO16 6YD UK; 13 Department of Pathology, Queen Elizabeth University Hospital, Glasgow, G51 4TF UK; 14 Department of Surgery, Queen Elizabeth University Hospital, Glasgow, G51 4TF UK

**Correction: J Exp Clin Cancer Res 43, 64 (2021)**

**https://doi.org/10.1186/s13046-024-02958-4**

Following publication of the article [1], the authors identified error in Figs. 6 M wherein incorrect image was pasted and processed.

The corrected figure is provided below.

Incorrect Fig. 6M

**Fig. 6:**
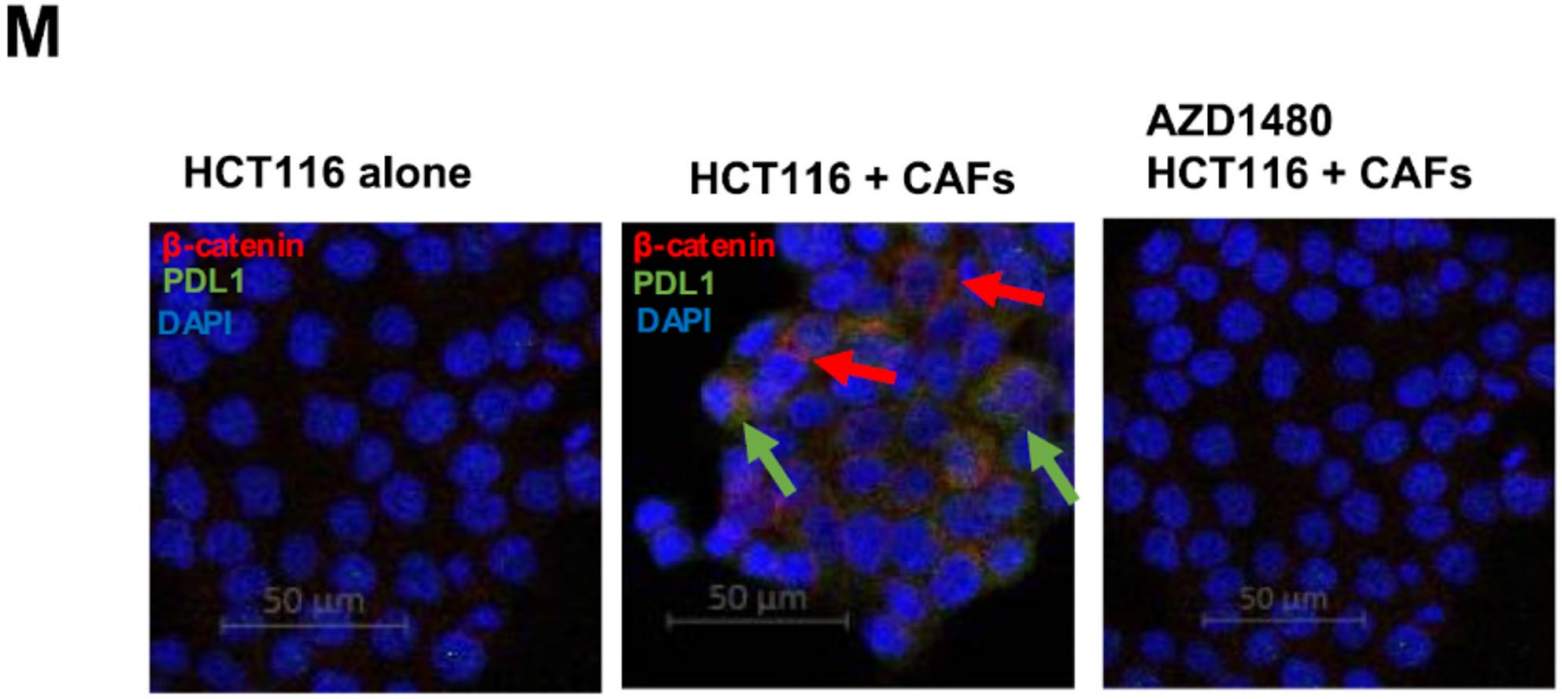
Activation of STAT3 in the tumor-associated stroma. Box plot showing the expression of STAT3 in different cell populations from a cohort of CRC patients from counfoundR (**A-B**). Enrichment plot showing IL6/JAK/STAT3 in a CRC cohort comparing epithelial and fibroblas t expression using confoundR (**C**). Box plot showing the expression of pSTAT3^tyr705^ within the tumor and stromal compartments of cohort 1 (**D**). Box plot showing the expression of pSTAT3^tyr705^ within the stroma relative to TSP status of cohort 1 (**E**). Kaplan Meier survival curve showing the association between stromal pSTAT3 and CSS in cohort 1 (**F**). Scatter plot showing the association between pSTAT3 expression within the tumor and stroma of cohort 1 (**G**). Kaplan Meier survival curve showing the association between a combined tumor and stromal score for pSTAT3^tyr705^ and CSS in cohort 1 (**H**). Brightfield images of untreated/AZD1480 treated CCD18Co colorectal fibroblasts (**I**). Bar chart showing the effect of AZD1480 on cell viability of CCD18Co cells (**J**) and a patient-derived CAF primary cell line (**K**). Representative images of IF staining for MHC1 in HCT116 cells grown as a monoculture and as a coculture with CAFs (**L**) Representative images of IF staining for β-catenin and programmed death ligand 1 (PDL1) in HCT116 cells grown as a monoculture, as a coculture with CAFs treated with vehicle control or AZD1480 (**M**). Significance set to *p* < 0.05*,* p* < 0.005**,* p* < 0.0005***,* p* < 0.00005****

Correct Fig. 6M

**Fig. 6:**
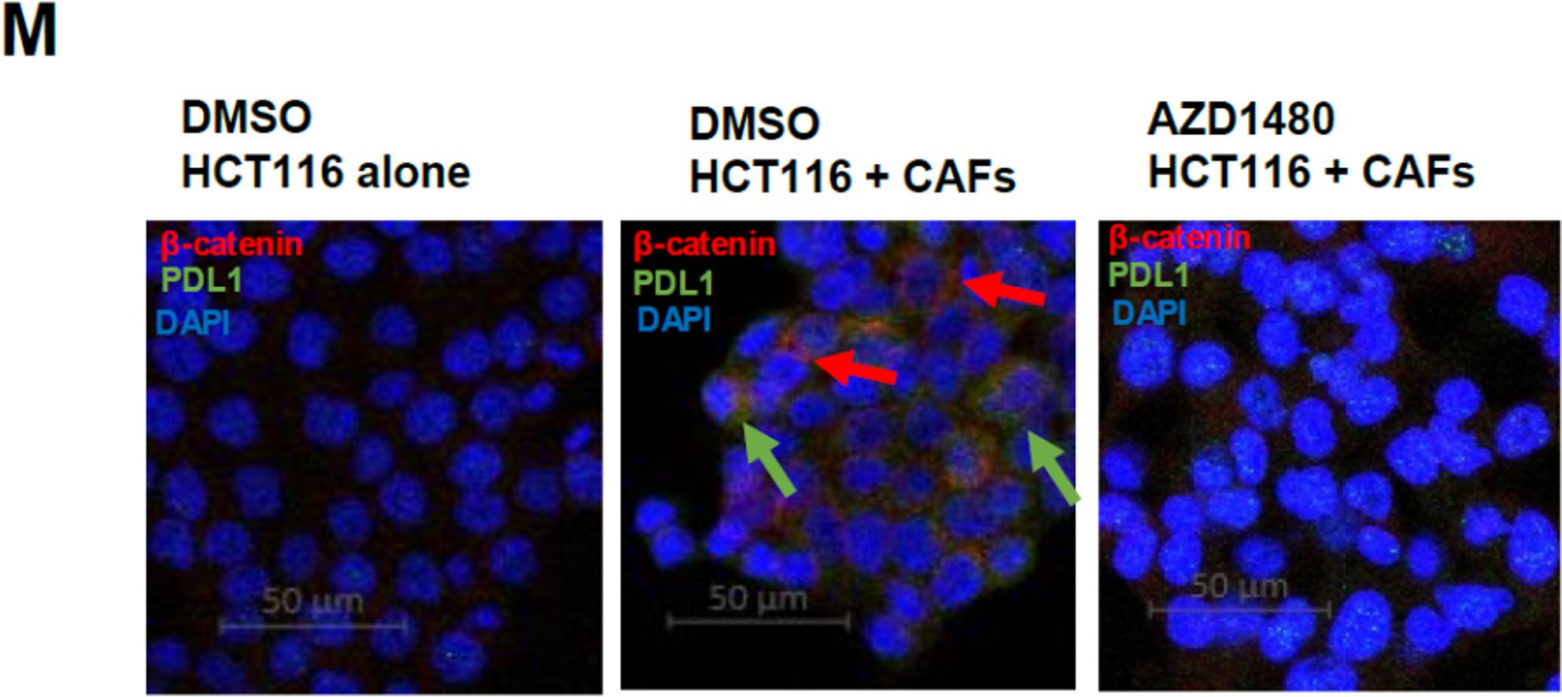
Activation of STAT3 in the tumor-associated stroma. Box plot showing the expression of STAT3 in different cell populations from a cohort of CRC patients from counfoundR (**A-B**). Enrichment plot showing IL6/JAK/STAT3 in a CRC cohort comparing epithelial and fibroblas t expression using confoundR (**C**). Box plot showing the expression of pSTAT3^tyr705^ within the tumor and stromal compartments of cohort 1 (**D**). Box plot showing the expression of pSTAT3^tyr705^ within the stroma relative to TSP status of cohort 1 (**E**). Kaplan Meier survival curve showing the association between stromal pSTAT3 and CSS in cohort 1 (**F**). Scatter plot showing the association between pSTAT3 expression within the tumor and stroma of cohort 1 (**G**). Kaplan Meier survival curve showing the association between a combined tumor and stromal score for pSTAT3^tyr705^ and CSS in cohort 1 (**H**). Brightfield images of untreated/AZD1480 treated CCD18Co colorectal fibroblasts (**I**). Bar chart showing the effect of AZD1480 on cell viability of CCD18Co cells (**J**) and a patient-derived CAF primary cell line (**K**). Representative images of IF staining for MHC1 in HCT116 cells grown as a monoculture and as a coculture with CAFs (**L**) Representative images of IF staining for β-catenin and programmed death ligand 1 (PDL1) in HCT116 cells grown as a monoculture, as a coculture with CAFs treated with vehicle control or AZD1480 (**M**). Significance set to *p* < 0.05*,* p* < 0.005**,* p* < 0.0005***,* p* < 0.00005****

This correction does not affect the results, interpretation, or conclusions of the article. The original article [1] has been corrected.
