# Supplementary figures and images for: JAK/STAT3 represents a therapeutic target for colorectal cancer patients with stromal-rich tumors
Source: J Exp Clin Cancer Res. 2024 Mar 1;43:64. doi: 10.1186/s13046-024-02958-4 (PMC10905886; doi:10.1186/s13046-024-02958-4)

A

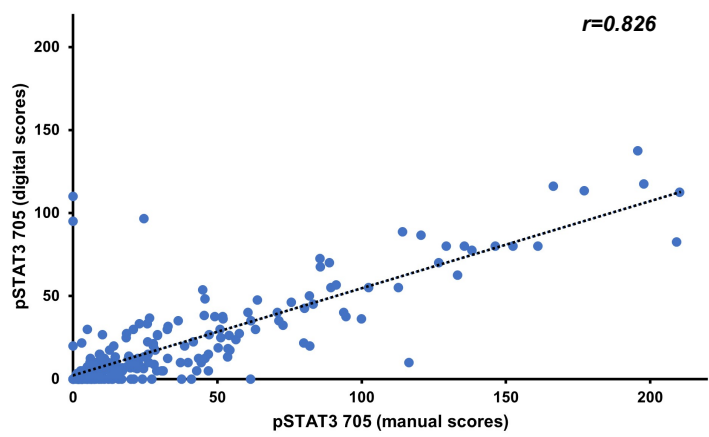

B

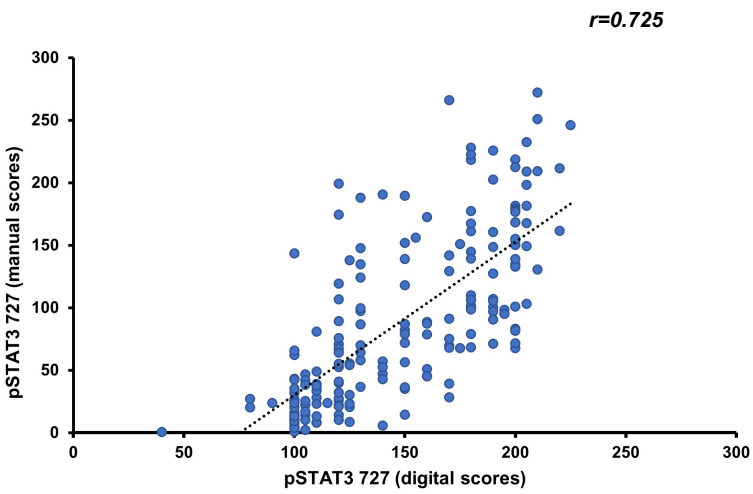

Supplement: Supplementary file 1 — Additional file 1: Figure S1. Validation of manual scoring using digital scoring. [file 13046_2024_2958_MOESM1_ESM.pdf]

**A**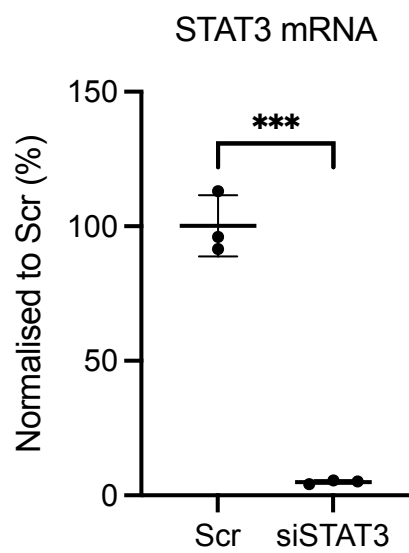**B**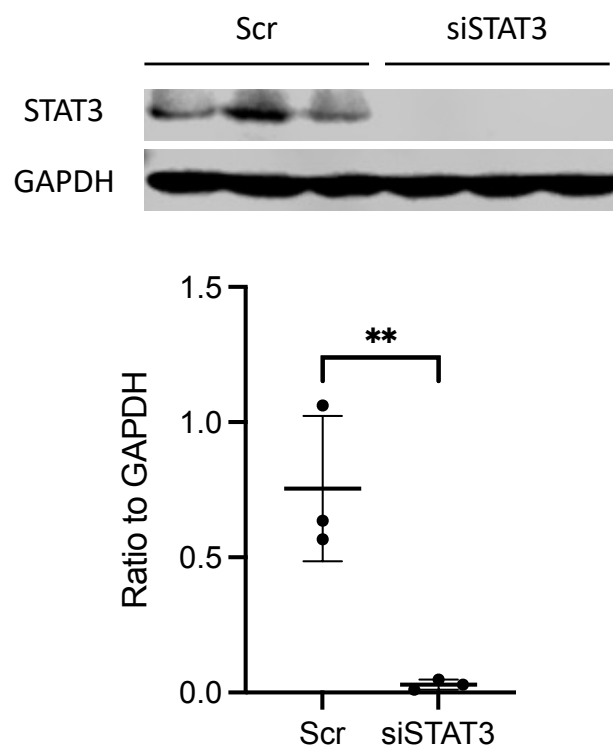

Supplement: Supplementary file 2 — Additional file 2: Figure S2. Validation of silencing STAT3 in HCT116. [file 13046_2024_2958_MOESM2_ESM.pdf]

A

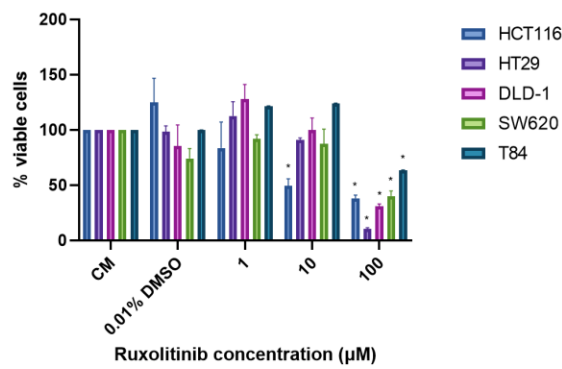

B

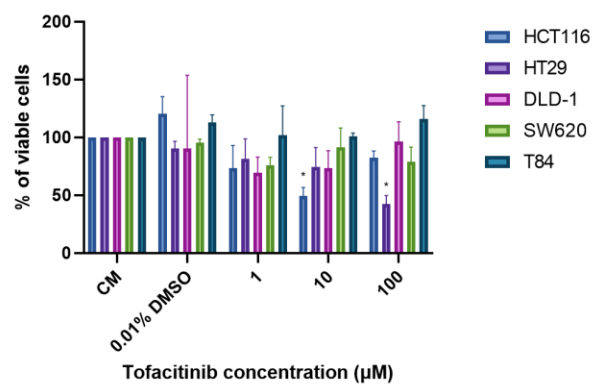

C

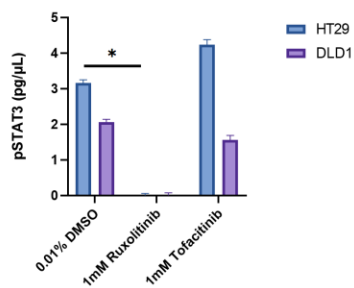

Supplement: Supplementary file 3 — Additional file 3: Figure S3. Assessment of JAK inhibitors in vitro. [file 13046_2024_2958_MOESM3_ESM.pdf]

A

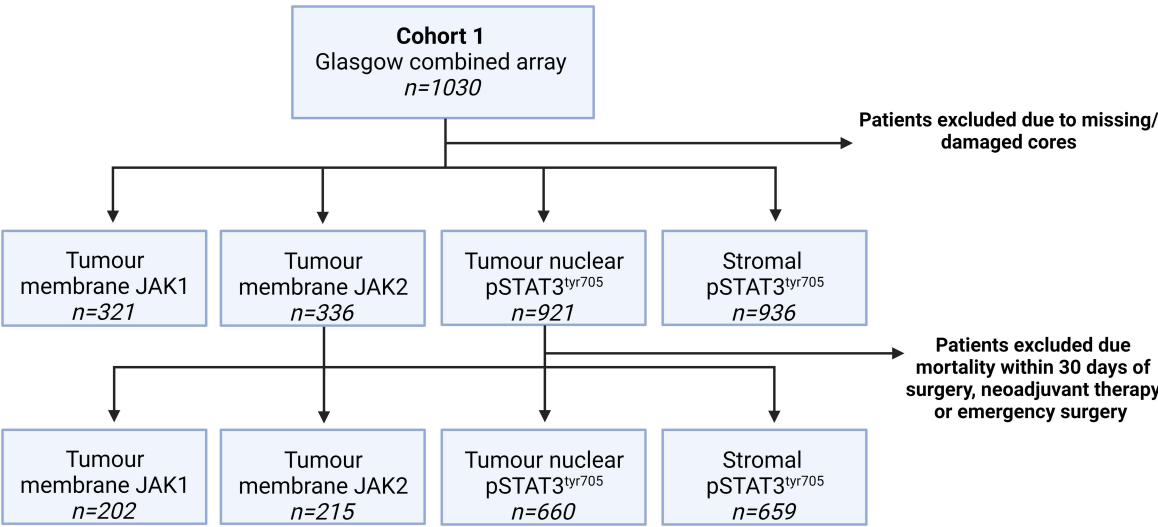

B

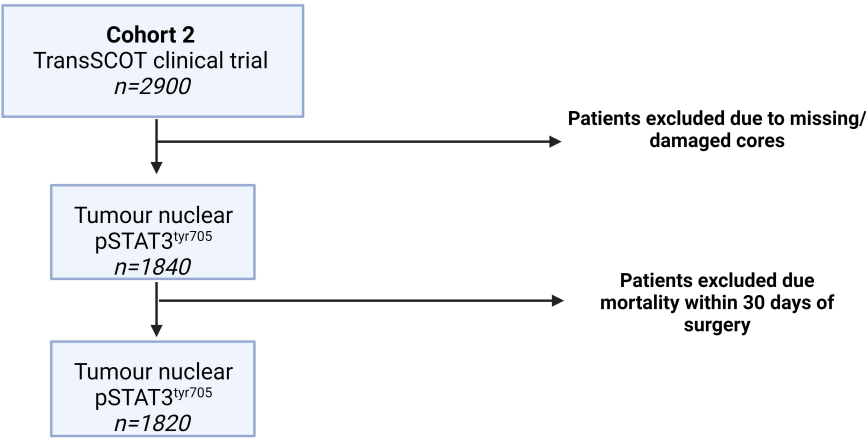

C

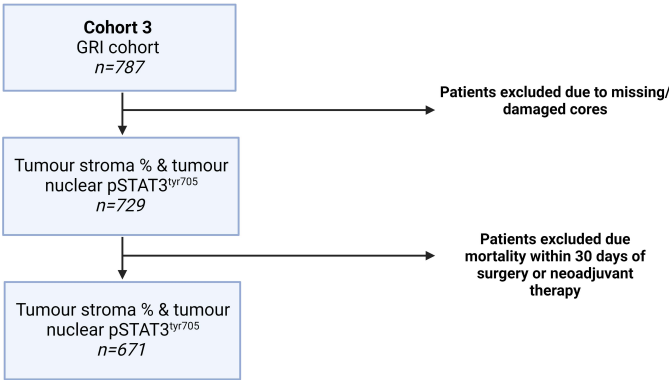

Supplement: Supplementary file 4 — Additional file 4: Figure S4. Patients included in analysis of retrospective cohorts. [file 13046_2024_2958_MOESM4_ESM.pdf]
