# Supplementary material for: JAK/STAT3 represents a therapeutic target for colorectal cancer patients with stromal-rich tumors
Source: J Exp Clin Cancer Res. 2024 Mar 1;43:64. doi: 10.1186/s13046-024-02958-4 (PMC10905886; doi:10.1186/s13046-024-02958-4)
Supplement: Supplementary file 6 — Additional file 6: Table S2. Chi-squared table of association between pSTAT3tyr705 and clinical features. [file 13046_2024_2958_MOESM6_ESM.docx]

| **Clinicopathological characteristic** | **Tumour pSTAT3^Tyr705^**  **Low (n=301) High (n=359)** | | **p** |
| --- | --- | --- | --- |
| **Age**  <65  >65 | 80 (26.6)  221 (73.4) | 128 (35.7)  231 (64.3) | **0.008** |
| **Sex**  Male  Female | 152 (50.5)  149 (49.5) | 174 (48.5)  185 (51.5) | 0.329 |
| **T stage**  I  II  III  IV | 22 (7.3)  51 (16.9)  168 (55.8)  60 (19.9) | 14 (3.9)  46 (12.8)  205 (57.1)  94 (26.2) | **0.042** |
| **N Stage**  0  I  II | 205 (68.8)  66 (22.1)  27 (9.1) | 215 (59.9)  106 (29.5)  38 (10.6) | 0.054 |
| **Tumour Subsite**  Right  Left  Rectum | 127 (42.6)  104 (34.9)  67 (22.5) | 142 (39.6)  107 (29.8)  110 (30.6) | 0.056 |
| **Tumour Differentiation**  Low  High | 270 (89.7)  31 (10.3) | 325 (90.5)  34 (9.5) | 0.410 |
| **Vascular Invasion**  Absent  Present | 208 (69.1)  93 (30.9) | 251 (69.9)  108 (30.1) | 0.443 |
| **KM Grade**  0-1  2-3 | 181 (60.7)  117 (39.3) | 231 (66.2)  118 (33.8) | 0.088 |
| **TSP**  Low  High | 246 (81.7)  55 (18.3) | 253 (74.6)  86 (25.4) | **0.019** |
| **Ki67 proliferation index**  <30%  >30% | 86 (28.6)  215 (71.4) | 225 (63.2)  131 (36.8) | **<0.001** |

**Supplementary Table 2:** Association between pSTAT3^tyr705^ expression and clinicopathological characteristics
