# Supplementary material for: JAK/STAT3 represents a therapeutic target for colorectal cancer patients with stromal-rich tumors
Source: J Exp Clin Cancer Res. 2024 Mar 1;43:64. doi: 10.1186/s13046-024-02958-4 (PMC10905886; doi:10.1186/s13046-024-02958-4)
Supplement: Supplementary file 7 — Additional file 7. Table S3. Univariate and multivariate cox regression on cohort 1. [file 13046_2024_2958_MOESM7_ESM.docx]

|  | Univariate | p | Multivariate | p |
| --- | --- | --- | --- | --- |
| Age  <65  >65 | 1.193 (0.882-1.615) | 0.247 | - | - |
| Sex  Male  Female | 1.163 (0.878-1.540) | 0.291 | - | - |
| T stage  1  2  3  4 | 1.848 (1.498-2.281) | <0.001 | 0.874 (0.597-1.279) | 0.487 |
| N stage  0  1  2 | 2.168 (1.813-2.592) | <0.001 | 1.773 (1.420-21.213) | <0.001 |
| Subsite  Right  Left  Rectal | 1.031 (0.868-1.224) | 0.728 | - | - |
| Peritoneal Involvement  Absent  Present | 2.602 (1.956-3.462) | <0.001 | 1.815 (1.291-2.552) | 0.001 |
| Vascular Invasion  Absent  Present | 2.211 (1.670-2.928) | <0.001 | 1.821 (1.284-2.583) | 0.001 |
| Margin Involvement  Absent  Present | 3.733 (2.393-5.821) | <0.001 | 2.486 (1.482-4.173) | 0.001 |
| Klintrup Makinen Grade  Low (0-1)  High (2-3) | 0.367 (0.256-0.527) | <0.001 | 0.425 (0.278-0.648) | <0.001 |
| Ki67 Status  <30%  >30% | 0.521 (0.389-0.696) | <0.001 | 0.733 (1.123-2.547) | 0.086 |
| TSP  <50%  >50% | 2.060 (1.525-2.782) | <0.001 | 1.466 (1.026-2.094) | 0.036 |
| Tumour pSTAT3  Low  High | 1.546 (1.131-2.113) | 0.006 | 0.936 (0.633-1.383) | 0.740 |
| Stromal pSTAT3  Low  High | 1.791 (1.257-2.554) | 0.001 | 1.691 (1.123-2.547) | 0.012 |
| Membrane JAK1  Low  High | 1.557 (0.798-3.036) | 0.194 | - | - |
| Membrane JAK2  Low  High | 1.590(0.751-3.366) | 0.226 | - | - |
